# Characterisation of a novel transcript LNPPS acting as tumour suppressor in bladder cancer via PDCD5‐mediated p53 degradation blockage

**DOI:** 10.1002/ctm2.1149

**Published:** 2022-12-28

**Authors:** Juan Li, Yifan Wang, Xinya Zhang, Xuemei Yang, Qiuchen Qi, Qi Mi, Maoxiao Feng, Yunshan Wang, Chuanxin Wang, Peilong Li, Lutao Du

**Affiliations:** ^1^ Department of Clinical Laboratory The Second Hospital Cheeloo College of Medicine Shandong University Jinan Shandong China; ^2^ Shandong Engineering & Technology Research Center for Tumor Marker Detection Jinan Shandong China; ^3^ Shandong Provincial Clinical Medicine Research Center for Clinical Laboratory Jinan Shandong China

**Keywords:** bladder cancer, lncRNA, m^6^A, ubiquitination

## Abstract

**Background:**

Long non‐coding RNAs (lncRNAs) play a crucial role in tumour initiation and progression. However, little is known about their contributions to p53‐related bladder cancer (BC) inhibition.

**Methods:**

By using high‐throughput sequencing, we screened the expression profiles of lncRNAs in BC and adjacent non‐tumour tissues. The roles of a novel lncRNA, named LNPPS [a lncRNA for programmed cell death 5 (PDCD5) and p53 stability], were determined by gain‐ and loss‐of‐function assays. RNA pull‐down followed by mass spectrometry analysis, RNA immunoprecipitation assays and other immunoprecipitation assays were performed to reveal the interactions among LNPPS, PDCD5 and p53, and the regulatory effect of LNPPS on the complex ubiquitination network comprising PDCD5, p53 and mouse double minute 2 homologue (MDM2).

**Results:**

LNPPS was downregulated in BC and markedly inhibited the viability of BC cells by inducing PDCD5/p53‐related apoptosis in vivo and in vitro. Mechanistically, LNPPS, serving as a scaffold, connected PDCD5 and p53 with nucleotides (nt) located at 121‒251 nt and 251‒306 nt of LNPPS, respectively. This process allowed LNPPS to protect PDCD5 from proteasomal degradation by blocking its K20 site ubiquitination. On the other hand, the increased interaction between PDCD5 and p53 displaced p53 from the MDM2‒p53 ubiquitination complex, resulting in an increase in p53 expression and related apoptosis levels. Moreover, LNPPS could induce the accumulation of PDCD5 and p53 in the nucleus and exert a synergistic effect on the prevention of protein degradation. In addition, we confirmed that the downregulation of LNPPS in BC was mediated by the decreased N6‐methyladenosine (m^6^A) modification.

**Conclusion:**

Our findings highlight a novel cross‐talk between LNPPS and the PDCD5/p53/MDM2 ubiquitination axis in BC development, indicating its potential as a therapeutic target for BC patients.

## Graphical abstract

1. A novel m^6^A‐regulated lncRNA LNPPS played a suppressive role in BC development.

2. LNPPS, a novel PDCD5‐interacting partner, protected PDCD5 from proteasome degradation by masking its K20 site ubiquitination.

3. LNPPS acted as a scaffold to connect PDCD5 with p53, impairing MDM2‐mediated p53 ubiquitinationb.

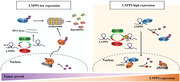

## 1 BACKGROUND

Bladder cancer (BC) is the fourth most frequently diagnosed malignancy worldwide, accounting for about 3.0% of new cancer cases diagnosed and 2.1% of cancer‐related deaths annually.^1^, ^2^ Such urological diseases range from recurrent non‐invasive tumours to aggressive and advanced‐stage tumours with high disease‐specific mortality.^3^ Because of the lack of effective therapeutic targets, BC patients commonly receive radical cystectomy in combination with chemotherapy or radiation therapy, resulting in very limited treatment efficiency.^4^, ^5^ In recent years, targeted therapies have been widely applied in clinical practice. However, such a regimen benefits only a small subgroup of BC patients.^6^ Thus, there is a pressing need to develop more favourable therapeutic targets and strategies.

lncRNAs are more than 200 nucleotides (nt), most of which lack the ability to encode proteins.^7^ Several studies have reported that lncRNAs are enriched in a tissue‐specific manner and emerge as crucial regulators in many pathological processes of cancer.^8^, ^9^ They can regulate the transcription, translation and post‐translation of oncogenes or tumour suppressors by acting as guides, decoys, scaffolds, competing endogenous RNAs, etc., ultimately leading to cancer reprogram.^10^ More recent evidence has indicated that scaffold lncRNAs act as central platforms for tethering proteins and further direct them to specific genomic locations or cellular structural domains to regulate biological processes in cancer.^11^, ^12^ For instance, a novel lncRNA termed Low expressed in Bladder Cancer Stem cells (lnc‐LBCS), which binds to hnRNPK and EZH2, and guides this complex to inhibit the transcription of SOX2, leading to enhanced chemoresistance for BC.^13^ Besides, a lncRNA, named lnc‐CCDST, has been reported to promote the degradation of DExH‐box helicase 9 (DHX9) by promoting the formation of the E3 ubiquitin ligase mouse double minute 2 homologue (MDM2)/DHX9 complex in cervical cancer.^14^ Nevertheless, the molecular mechanisms of such lncRNA‐mediated regulation in BC have been merely the tip of the iceberg.

It has been shown that p53 is the hub regulator of the cellular signalling network.^15^ As one of the most prominent outcomes of p53 activation, p53‐induced apoptosis is found to be tightly controlled by programmed cell death 5 (PDCD5) in hepatocellular carcinoma.^16^ Recent studies have shown that PDCD5 stabilises p53 by mediating the separation of MDM2 from p53, which hampers the ubiquitin‒proteasome degradation of p53.^17^, ^18^ This also raises the possibility that p53 is recruited to the promoter of pro‐apoptotic targets such as BCL2‐associated X protein (BAX) and p53 up‐regulated modulator of apoptosis (PUMA).^19^ Currently, the molecular mechanisms of PDCD5/p53‐associated apoptosis in BC cells are poorly understood, particularly the roles of lncRNAs in this process.

In this study, based on RNA‐seq data from BC tissues and the gain‐ and loss‐of function assays, we identified a novel tumour‐suppressive lncRNA, named LNPPS (a lncRNA for PDCD5 and p53 stability, ENST00000622374). Further investigations showed that the downregulation of LNPPS was regulated by N6‐methyladenosine (m^6^A) modification in BC. Mechanistically, LNPPS acted as a scaffold for PDCD5 and p53, blocking the K20 site ubiquitination of PDCD5 and disrupting MDM2‐mediated p53 ubiquitination, which promoted p53‐related cell apoptosis. Our findings provide novel insights into lncRNA‐related protein ubiquitination in BC and contribute to the identification of potential therapeutic targets.

## 2 MATERIALS AND METHODS

### 2.1 Patients’ tissues specimens

BC tumour tissues and adjacent non‐tumour tissues were derived from patients undergoing surgery at The Second Hospital of Shandong University in 2017–2020. Patients did not receive radiotherapy or chemotherapy before surgery, and were classified as having BC based on the seventh edition of the American Joint Committee on Cancer staging manual. Tissues were washed in phosphate‐buffered saline (PBS) buffer, frozen in liquid nitrogen for 30 min, and subsequently stored at –80°C. This work was approved by the Ethics Committee of The Second Hospital of Shandong University.

### 2.2 Cell culture and transfection

5637, J82, UM‐UC‐3, T24, SV‐HUC‐1, RT4, NCI‐H1299, A549, MDA‐MB‐468, MDA‐MB‐231, MCF‐7, T‐47D, SW116, SW620 and HEK293T were acquired from the Cell Bank of the Chinese Academy of Sciences (Shanghai, China), tested negative for mycoplasma and incubated at 37°C with 5% CO_2_. 5637, T24, NCI‐H1299, T‐47D and A549 were cultured in Roswell Park Memorial Institute 1640 medium (Gibco). J82, UM‐UC‐3 and HEK293T were cultured in Dulbecco's modified Eagle's medium (Gibco). SW116, SW620, MDA‐MB‐468 and MDA‐MB‐231 were cultured in Leibovitz's L‐15 medium (Gibco). RT4, SV‐HUC‐1 and MCF‐7 were cultured in McCoy's 5A (Gibco), Ham's F‐12K medium (Macgene, Beijing, China) and Minimum Essential Medium (Macgene), respectively. Medium was added with 10% foetal bovine serum (FBS) (Sagecreation, Beijing, China) and 1% penicillin/streptomycin (Solarbio, Beijing, China).

The transfection of plasmids and siRNAs was performed with Lipofectamine 2000 (Invitrogen, USA) based on the manufacturer's protocols. Lentivirus overexpressing LNPPS or the CRISPR‐dcas9‐KRAB/sgRNAs for silencing LNPPS were transfected into BC cells with 5 mg/ml polybrene for 18 h. Cells were then selected by puromycin (2 μg/ml) or blasticidin S (1 μg/ml). Detailed descriptions of siRNAs and sgRNAs are shown in Table S1.

### 2.3 RNA extraction and quantitative polymerase chain reaction with reverse transcription

Total RNA was extracted from tissues or cells by using TRIzol Reagent (Life Technologies, USA) or RNA Fast2000 Reagent (Fastagen, China), respectively. PrimeScript RT Reagent Kit and TB Green Premix Ex Taq (Takara, Dalian, China) were used for cDNA synthesis and quantitative application, respectively. The relative levels of genes were analysed by the 2^−ΔΔCT^ method [Glyceraldehyde‐3‐phosphate dehydrogenase (GAPDH) was used as the endogenous control]. All primer sequences are shown in Table S2.

### 2.4 Western blotting/immunoblotting analysis

Proteins were extracted from indicated cells by radio‐immunoprecipitation assay (RIPA) lysis buffer containing protease inhibitors (Roche, Indianapolis, IN, USA). The samples were separated by sodium dodecyl sulfate‐polyacrylamide gel electrophoresis (SDS‐PAGE) gels and transferred to poly(vinylidene fluoride) membranes (Millipore, Germany). After blocking with 5% bovine serum albumin (BSA) buffer, membranes were incubated with primary antibodies overnight at 4°C and subsequently incubated with horseradish peroxidase‐labelled secondary antibodies (CST, 7074 or #076). Finally, signals from blots were determined by the enhanced chemiluminescence system (Bio‐Rad). The primary antibodies are listed in Table S3.

### 2.5 Subcellular RNA and protein fractionation assays

Following the manufacturer's protocols, subcellular fractions of RNA and protein were separated and purified with the PARISTM Kit Protein and RNA Isolation System (Thermo Fisher Scientific, Waltham, MA, USA). RNAs extracted from different fractions were performed with quantitative reverse transcription‐polymerase chain reaction (qRT‐PCR) using GAPDH and U6 as markers of the cytoplasm and nucleus, respectively. Similarly, the expression of proteins in subcellular fractionations was measured by Western blotting (WB) analysis. The anti‐β‐actin antibody and anti‐LaminB1 antibody specifically marked the cytoplasm and nucleus, respectively.

### 2.6 RNA fluorescence in situ hybridisation assays and immunofluorescence assays

The indicated cells were placed on the culture slides before assays. After three washes with PBS, the slides were fixed using 4% paraformaldehyde, permeabilised with 0.5% Triton X‐100 at 4°C, blocked in pre‐made hybridisation buffer at 37°C, and incubated in hybridisation buffer containing RNA probes (RiboBio, Guangzhou, China) overnight at 37°C. Next, the slides were rinsed with citric acid–sodium citrate buffer, stained with 4′,6‐diamidino‐2‐phenylindole (DAPI) and rinsed another three times. Finally, images of the slides were taken by the confocal imaging system (LSM 780, Carl Zeiss, Jena, Germany). For immunofluorescence (IF) assays, slides were briefly washed, fixed and permeabilised as described above. After blocking in 3% BSA, slides were first incubated with specific primary antibodies overnight at 4°C and then incubated with Alexa488‐ or Alexa555‐labelled secondary antibodies in the dark. After being stained with DAPI and another wash, slides were observed under a confocal imaging system (LSM 780). The species of primary antibodies should be different in double IF assays.

### 2.7 Immunohistochemistry staining

After being fixed with 4% paraformaldehyde, tumour tissues from mice were made into paraffin‐embedded slides. Slides were washed in xylene, rehydrated via serial dilutions of alcohol, and incubated in H_2_O_2_ to remove endogenous catalase. After being blocked and incubated with specific primary antibodies, slides were treated with the MaxVision TMHRP‐Polymer anti‐rabbit immunohistochemistry (IHC) kit (MXB Biotechnologies, Fujian, China), stained with 3,3‐diaminobenzidine tetra‐hydrochloride and counter‐stained with haematoxylin. The slides were captured by an Olympus BX51 microscope and quantified by the histological score (*H*‐score). *H*‐score = ∑(*pi* × *i*) = (percentage of weak intensity × 1) + (percentage of moderate intensity × 2) + (percentage of strong intensity × 3).

### 2.8 Cell growth, CCK8 assays, colony formation assays

After transfected with lentivirus or indicated sets of plasmids/siRNA, cell dynamic growth was obtained by an xCELLigence RTCA DP instrument (ACEA Biosciences, San Diego, CA, USA). For CCK8 assays, indicated cells were plated into 96‐well plates (2 × 10^4^ cells/well) in advance and measured every 24 h. Before detection, cells were incubated with CCK8 reagent (BestBio, Shanghai, China) following the manufacturer's protocols. Cell viability was evaluated by OD values (450 nm) with a microplate reader (Molecular Devices, USA). For colony formation assays, 1 × 10^3^ cells were plated into the six‐well plates. Colonies were fixed in methanol and stained with 1% crystal violet (Solarbio) after 2 weeks of culture.

### 2.9 Transwell assays

A total of 5 × 10^4^ or 8 × 10^4^ cells in 200 μl medium (serum‐free) were added to the upper chambers of inserts (8‐μm pore size; Corning) for migration or invasion assays, respectively. Then, 800 μl medium (20% FBS) was added to the lower chambers. After the 24 or 48 h of culture, inserts were washed, fixed in methanol and stained with Giemsa (Solarbio), and the cells in the upper chambers of the inserts were removed with cotton swabs. The images were taken under a microscope (Zeiss, Axio Observer). For invasion assays, the inserts (upper chamber) should be pre‐coated with Matrigel (BD Biosciences, San Jose, CA, USA).

### 2.10 Cell cycle and cell apoptosis assays

The indicated cells were re‐suspended to obtain single‐cell suspensions in advance. For cell cycle assays, cell suspensions were fixed with appropriate amount of ethanol at –20°C, stained with propidium iodide containing RNase A (BestBio) and finally subjected to the flow cytometry (BD Biosciences). For apoptosis assays, cells were treated with the Annexin V‐FITC/PI or Annexin V‐APC/PI apoptosis detection kits (BestBio). ModFit LT software (Verity Software House, USA) and FlowJo software (Tree Star, Inc.) were used for the further analysis of cell cycle and cell apoptosis, respectively.

### 2.11 RNA pull‐down with mass spectrometry analysis

The sense, antisense and truncated sequences were obtained by in vitro transcription using the Riboprobe Combination System‐T3/T7 Kit (Promega, Carlsbad, CA, USA) and labelled with desthiobiotinylate by the Pierce RNA 3′ End Desthiobiotinylation Kit (Thermo Fisher Scientific). Protein lysates were obtained from indicated cells using IP lysis buffer containing protease inhibitors. The RNA pull‐down assays were then carried out with the Pierce Magnetic RNA‐Protein Pull‐Down Kit (Thermo Fisher Scientific). The precipitated proteins were subjected to mass spectrometry (MS), which was further visualised by silver staining and WB. In advance of MS, proteins were digested with trypsin (Promega, V5113), desalted, and concentrated using C18‐based solid phase extraction. Peptides were further analysed by using high resolution/high mass accuracy reversed phase (18) nano‐liquid chromatography–mass spectrometry (LC–MS)/MS (Orbitrap elite, Thermo Fisher Scientific).

### 2.12 RNA immunoprecipitation assays

The assays were performed by the EZ‐Magna RNA immunoprecipitation (RIP) RNA Binding Protein Immunoprecipitation Kit (Millipore, Bedford, MA, USA). In short, magnetic beads coated with indicated primary antibodies were incubated with the indicated cell lysates overnight. The immunoprecipitated RNA samples were purified and subjected to qRT‐PCR. The levels of relative enrichment were normalised to 10% RNA input.

### 2.13 m^6^A methylated RNA immunoprecipitation assays

m^6^A methylated RNA immunoprecipitation (MeRIP) assays were performed with the Manga MeRIP m^6^A Kit (Millipore, Bedford, MA, USA). In short, total RNA from cells was fragmented into 100 nt or smaller fragments using fragmentation buffer followed by magnetic immunoprecipitation (IP) with indicated primary antibody‐conjugated protein A/G beads. After being washed and purified by the RNeasy Mini Elute Cleanup Kit (QIAGEN, Germany), the isolated RNA fragments were subjected to qRT‐PCR. Ten percent of RNA fragments before IP were served as input to normalise m^6^A enrichment.

### 2.14 Immunoprecipitation and co‐immunoprecipitation assays

After being transfected, protein lysates were obtained from the indicated cells by RIPA buffer (weak) with protease inhibitors and were immunoprecipitated with indicated primary antibodies overnight at 4°C. Immune complexes were captured with magnetic Protein A/G agarose beads (Santa Cruz, CA, USA) for 2–4 h. The immunoprecipitated proteins were analysed by immunoblotting (IB) blots after washing. For ubiquitination assays, cells were treated with 10 μM MG132 (Sigma–Aldrich, USA) for 6 h before IP assays.

### 2.15 RNA‐seq, functional enrichment analysis and gene set enrichment analysis

The RNeasy mini kit was used to extract total RNA from the indicated tissues and cells (Qiagen, Germany). TruSeq RNA Sample Preparation Kit (Illumina, USA) was used to create paired‐end libraries that were then sequenced on the Illumina NovaSeq 6000 (Illumina). Gene abundance was presented as fragments per kilobase of exon per million reads mapped (FPKM). R package ‘edgeR’ was used to screen differentially expressed genes. For functional enrichment analysis, such as Gene Ontology (GO) enrichment and Kyoto Encyclopedia of Genes and Genomes (KEGG) pathway enrichment, was performed via the ‘enrich’ R package. Gene set enrichment analysis (GSEA) was performed by using the GSEA v2.0 tool.

### 2.16 Dual‐luciferase reporter assay

Cells overexpressing LNPPS plasmid or control empty vector were transfected by mixing either PG13‐luc [including 13 copies of wild‐type (WT) p53‐binding consensus sequence, Addgene], MG15‐luc (including 15 copies of mutated p53‐binding consensus sequence, Addgene) or an empty vector without both WT and mutated p53‐binding sequence with pRL‐TK plasmid (Promega) in a 100:1 ratio using the Lipofectamine 2000 (Invitrogen). After the 48 h of culture, the luciferase activities of cells were evaluated in a Dual‐Glo Luciferase reporter assay system (Promega) in a Glomax 96 (Promega). The relative luciferase activities were normalised to Renilla luciferase activities.

### 2.17 Chromatin immunoprecipitation assays

Protein A and G Dynabeads (88845, 88847, Thermo Fisher Scientific) were mixed and incubated with primary antibodies [chromatin immunoprecipitation (ChIP) grade, 5 μg for each sample] for 3 h. The indicated cells were immediately cross‐linked with 1% formaldehyde for 10 min and 125 mM glycine was quickly added to terminate the cross‐link. According to the study by Guo et al.,^20^ nuclear fractions of cells were extracted and sonicated after three washes. The 10% supernatant was taken and used as the DNA input. The remaining lysate was added to the antibody‐conjugated beads. After the overnight incubation, the beads were washed and reversed cross‐linked at 65°C. Finally, immunoprecipitated DNA samples were purified by the ChIP DNA Clean & Concentrator Kit (Zymo Research, USA) and subjected to qPCR. The primers for ChIP–qPCR are listed in Table S2.

### 2.18 In vivo assays

BALB/c nude mice (4‐week‐old male) were obtained from Beijing Vital River Laboratory Animal Technology (China) and were randomly placed in different groups (5 mice/group). A total of 5 × 10^6^ LNPPS stably overexpressed or knockdown 5637 cells and corresponding control cells were injected into the right flank of mice, respectively. After 2 weeks post‐injection, the volume of tumours was measured and calculated (volume = 0.5 × width^2^ × length). Mice were euthanised by carbon dioxide inhalation after 7 weeks, and the subcutaneous tumours were dissected and weighed. The in vivo assays were approved by the Ethics Committee of the Second Hospital of Shandong University.

### 2.19 Statistical analysis

Statistical analyses were assessed and viewed by SPSS 18.0 (Chicago, IL, USA) or GraphPad Prism5 (La Jolla, CA, USA). The data between the two groups were compared by the Student's *t*‐test. The Mann–Whitney *U*‐test was used when the population did not have a normal distribution. One‐way analysis of variance was performed for the analysis of variance in multiple groups. The correlation between the expression levels of LNPPS and m^6^A regulators [methyltransferase‐like 3 (METTL3)/methyltransferase‐like 14 (METTL14)/AlkB homologue 5 (ALKBH5)/fat mass and obesity‐associated protein (FTO)], LNPPS and PDCD5 or p53, and PDCD5 and p53 was analysed using Spearman correlation tests. All experiments were performed in at least three replicates. Quantitative data are shown as mean ± standard error of mean. Statistical significance was defined as a *p*‐value < .05.

## 3 RESULTS

### 3.1 LNPPS is a candidate lncRNA relevant to BC

**FIGURE 1:**
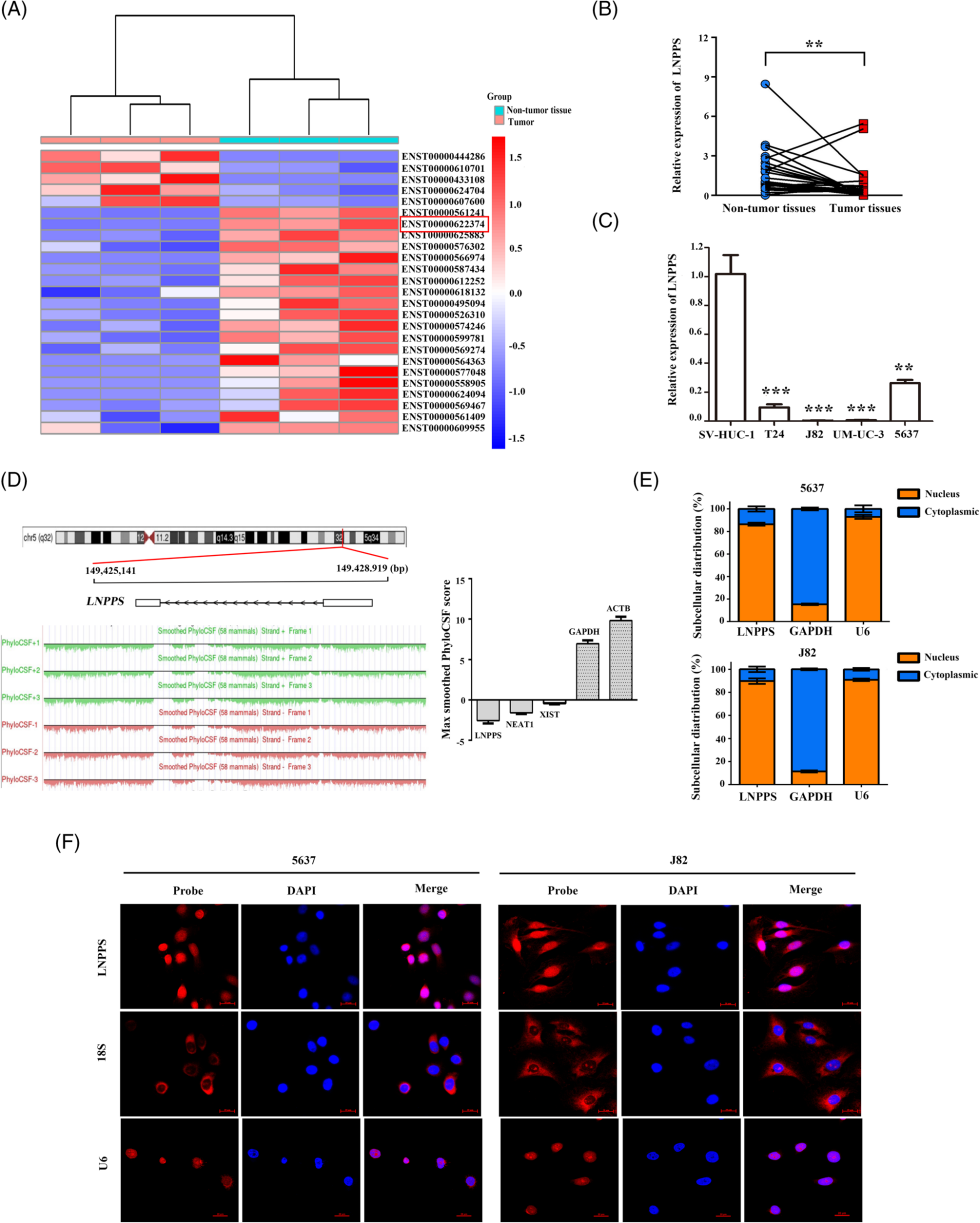
Long non‐coding RNA (lncRNA) LNPPS is downregulated in bladder cancer (BC). (A) The cluster heatmap of top 25 differently expressed lncRNAs in BC tissues compared with adjacent non‐tumour tissues (excluding two pairs of poor biological replicate samples). Red and blue colours indicated high and low expression levels relative to global median, respectively. (B) The expression of LNPPS in 27 pairs of BC tissues and adjacent non‐tumour tissues by quantitative reverse transcription‐polymerase chain reaction (qRT‐PCR). (C) The expression of LNPPS in multiple BC cell lines and immortalised human urothelial cell line (SV‐HUC‐1) by qRT‐PCR. (D) The chromatin signature and encoding substitution frequency analysis predicted by UCSC Genome Browser. Left: the chromatin location and PhyloCSF status at locus of LNPPS. Right: the encoding‐potential analysis by PhyloCSF. LncRNA X‐inactive specific transcript (XIST) and lncRNA nuclear‐enriched abundant transcript 1 (NEAT1): control non‐coding RNAs, GAPDH and Beta‐actin (ACTB): control encoding genes. (E) Cellular fractionation PCR showing the majority of LNPPS were in the nucleus. GAPDH: cytoplasmic control; U6: nucleus control. (F) RNA fluorescence in situ hybridisation (FISH) assays confirmed that LNPPS was mainly distributed in the nucleus of 5637 (left) and J82 (right) cells. Blue: 4′,6‐diamidino‐2‐phenylindole (DAPI); red: Cy3‐labelled LNPPS, 18s and U6 probes. 18s: cytoplasmic control. U6: nucleus control. All scale bars: 20 μm. ^*^ *p* < .05, ^**^ *p* < .01 and ^***^ *p* < .001

To identify potential functional lncRNAs required for BC development, the lncRNA expression profiles of tumour tissues and adjacent non‐tumour tissues from five BC patients were examined using RNA‐sequencing (raw data accessible via GSE190079). The heatmap depicted the 25 differently expressed lncRNAs (*p* < .05, log_2_|fold change| > 2.0) (Figure 1A and Table S4). According to the *p*‐values and FPKM, the top five upregulated and downregulated lncRNAs were screened for further confirmation. qRT‐PCR results showed that only lncRNA AC131025.2 (Ensemble: ENST00000622374, renamed as LNPPS) was significantly downregulated in BC tissues compared with paired non‐tumour tissues, consistent with the RNA‐seq data (Figures 1B and S1A–I).

LNPPS is located on human chromosome 5 at 149 425 771‒149 428 289 and is composed of two exons with a transcript length of 422 nt. We verified the full‐length sequence of LNPPS by PCR amplification with a series of gene‐specific primers in BC cells (Figure S1J). The PyhloCSF score of LNPPS was –2.42, and it was classified as a non‐coding sequence similar to other well‐known lncRNAs (Figure 1D). Consistently, the non‐coding nature of LNPPS was confirmed by ORF Finder, CPAT and CPC2 databases (Figure S1K–M). Furthermore, qRT‐PCR showed that LNPPS was significantly downregulated in all measured BC cells compared with SV‐HUC‐1 cells (Figure 1C). Additionally, the relative expression levels of LNPPS in BC cell lines were lower than those in non‐BC cell lines (Figure S1N). To determine the subcellular localisation of LNPPS, nuclear and cytoplasmic fractions of BC cells were extracted. qRT‐PCR demonstrated that LNPPS was mainly localised in the nucleus (Figures 1E and S1O). With RNA fluorescence in situ hybridisation (FISH) assays, LNPPS was also observed mainly in the nucleus (Figures 1F and S1P). Collectively, LNPPS is a lncRNA and is expressed at low level in BC.

### 3.2 LNPPS inhibits the viability of BC cells and enhances apoptosis

**FIGURE 2:**
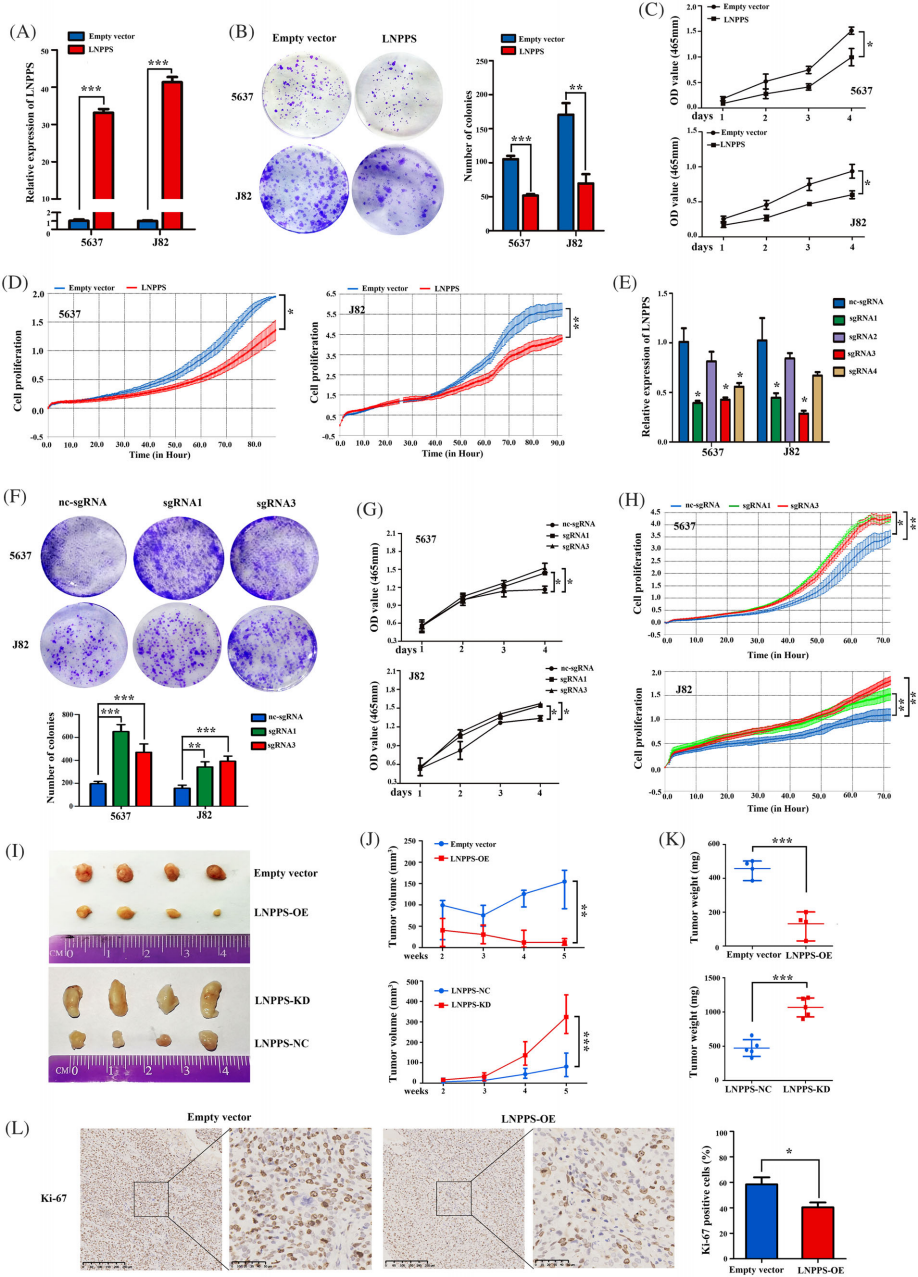
LNPPS inhibits bladder cancer (BC) cell viability in vitro and in vivo. (A and E) The overexpression efficiency (A) or knockdown efficiency (E) of LNPPS after transfected with its overexpression lentivirus or dCas9‐KRAB and specific sgRNAs lentivirus for silencing LNPPS in cells. (B and F) Colony formation assays on 5637 and J82 cells after LNPPS overexpression (B) or knockdown (F). (C and G) CCK8 assays on BC cells after LNPPS overexpression (C) or knockdown (G). (D and H) The cell growth dynamics of BC cells after LNPPS overexpression (D) or knockdown (H) by xCELLigence system. (I–K) Representative images of excised tumours (I), the growth curves of tumour volume (J) and final tumour weight (K) from LNPPS stably overexpressed and knockdown xenograft models, respectively. (L) The levels of Ki‐67 in xenograft tumours from LNPPS stably overexpressed mice models. Left: representative images. Right: quantisation of the percentage of Ki‐67‐positive cells. Scale bars: 250 and 50 μm, respectively. ^*^ *p* < .05, ^**^ *p* < .01 and ^***^ *p* < .001

To explore the roles of LNPPS in BC progression, we transfected lentiviral‐LNPPS and its control vector to construct BC cells that stably overexpressed LNPPS (Figure 2A). As indicated in colony formation assays, CCK8 assays and RTCA xCELLigence assays, overexpression of LNPPS inhibited BC cell proliferation (Figure 2B–D). Moreover, transwell assays demonstrated that the migration and invasion abilities of cells were reduced in LNPPS‐overexpressing cells (Figure S2A,B). Considering that most LNPPS was localised in the nucleus, a CRISPR interference system was used to knockdown LNPPS in BC cells. qRT‐PCR showed that sgRNA1 and sgRNA3 exhibited better silencing efficiency (Figure 2E). Consistent with the results of LNPPS overexpression, LNPPS knockdown promoted the proliferation, migration and invasion of BC cells (Figures 2F–H and S2C,D). The effects of LNPPS dysregulation on tumourigenicity were further investigated in nude mice. Similar to the findings in vitro, overexpression of LNPPS suppressed the growth of subcutaneous tumours, whereas LNPPS knockdown increased the tumour volume and weight (Figure 2I–K). IHC staining data of the subcutaneous tumours demonstrated that the proportion of Ki‐67‐positive cells was decreased by overexpression of LNPPS (Figure 2L).

**FIGURE 3:**
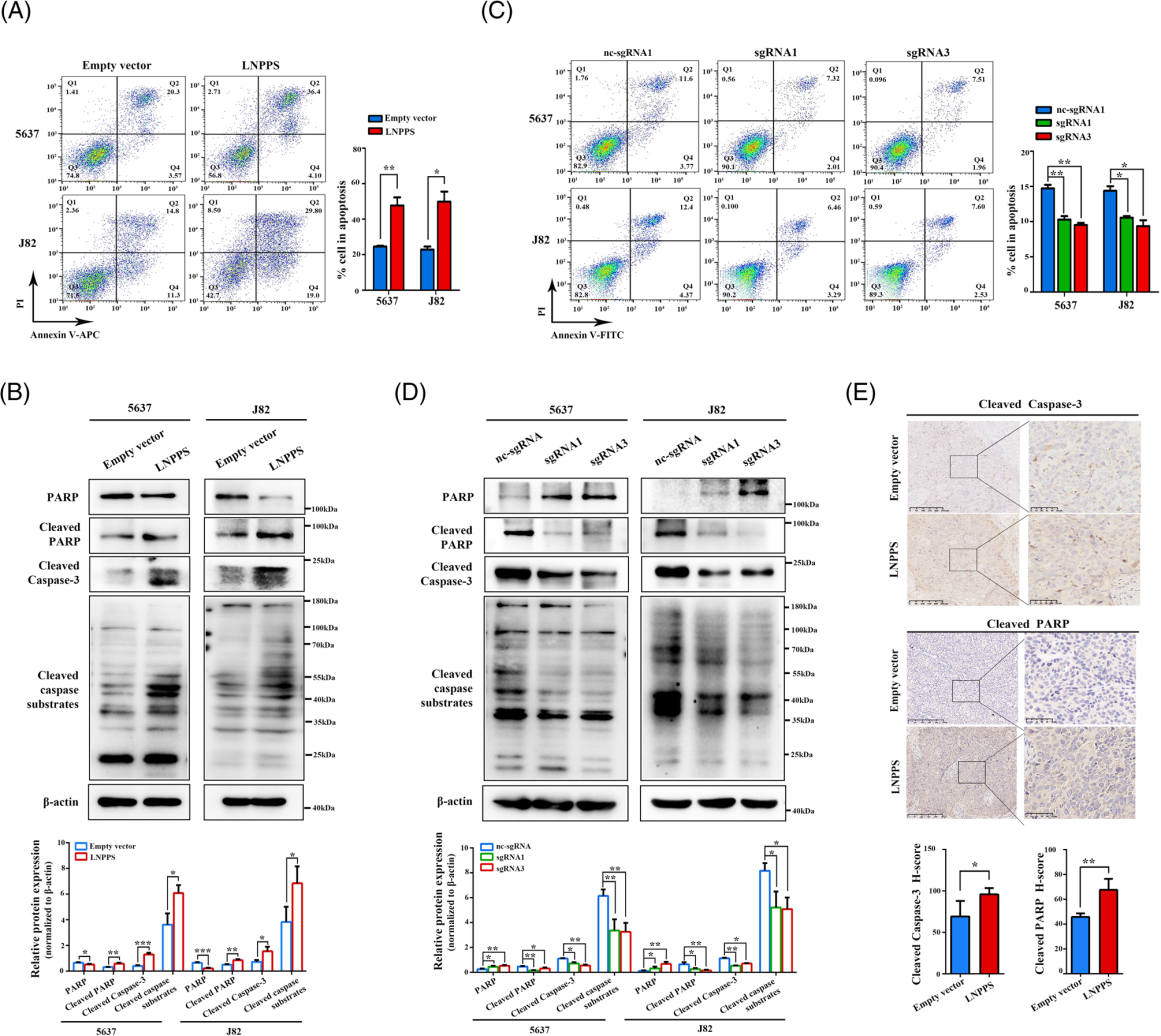
LNPPS enhances bladder cancer (BC) cells apoptosis in vitro and in vivo. (A and C) The effect of LNPPS overexpression (A) or knockdown (C) on the cell apoptosis of 5637 and J82 cells by flow cytometry. Left: representative images. Right: quantisation of the apoptotic rates of indicated cells. (B and D) Western blotting analysis of apoptosis‐related proteins in LNPPS overexpression (B) or knockdown (D) cells. (E) Representative images of immunohistochemistry (IHC) staining of cleaved caspase‐3 and cleaved PARP in paraffin‐embedded xenograft tumours from LNPPS stably overexpressed mice models. Scale bars: 250 and 50 μm, respectively. ^*^ *p* < .05, ^**^ *p* < .01 and ^***^ *p* < .001

Furthermore, cell apoptosis analysis indicated that the percentage of apoptotic cells increased upon LNPPS overexpression, which was further confirmed by WB analysis of apoptosis‐related proteins (Figure 3A,B). In contrast, silencing of LNPPS exhibited the opposite effects (Figure 3C,D). We next found that LNPPS overexpression had no significant effect on the proportion of cells in the G1, S and G2 phases (Figure S2E). The expressions of cell cycle‐related proteins, such as p21 and cyclinD1, were not apparently affected by LNPPS overexpression in BC cells (Figure S2F). Moreover, IHC assays showed that the expression of cleaved caspase‐3 and cleaved poly(ADP‐ribose) polymerase (PARP) was also increased in the subcutaneous tumours overexpressing LNPPS compared with the control group (Figure 3E). These findings indicate that LNPPS acts as a tumour suppressor that inhibits cell proliferation and promotes cell apoptosis in BC.

### 3.3 LNPPS specifically interacts with PDCD5

**FIGURE 4:**
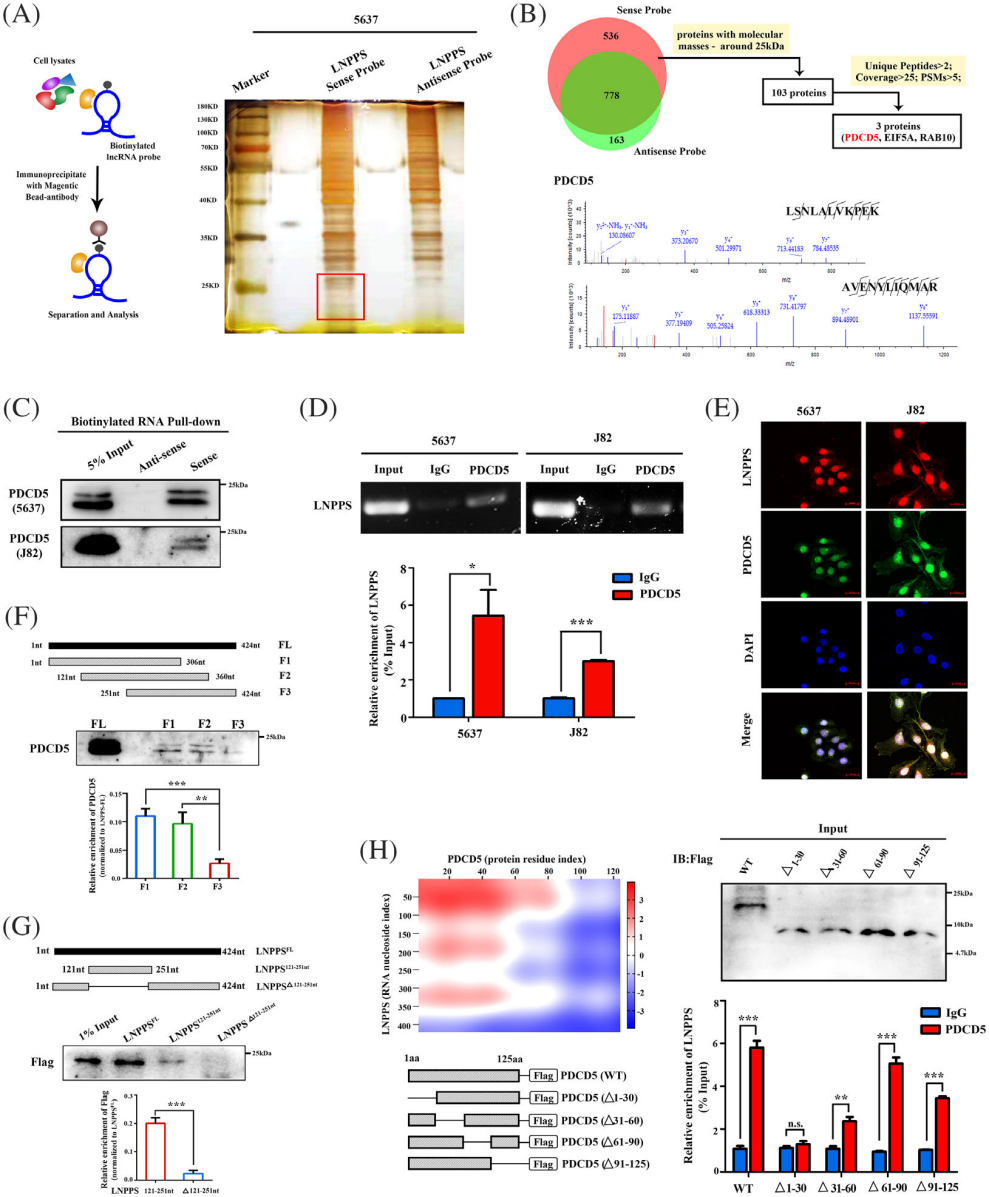
LNPPS specifically binds to programmed cell death 5 (PDCD5). (A) Identification of candidate LNPPS‐binding proteins by RNA pull‐down assays. Left: experimental flowchart of RNA pull‐down assays. Right: representative images of silver staining after incubation of biotin‐labelled sense or anti‐sense LNPPS probes with protein lysates from 5637 cells. Red box shows the major differential bands. (B) Upper: flowchart for screening out candidate LNPPS‐binding proteins by RNA pull‐down/mass spectrometry analysis. Lower: spectrums of peptides identified by mass spectrometry. (C) RNA pull‐down assays followed by Western blotting analysis showing the interaction between PDCD5 and LNPPS in 5637 and J82 cells. Note: low band is special for PDCD5. (D) RNA immunoprecipitation (RIP) assays in 5637 and J82 cells with anti‐PDCD5 and anti‐immunoglobulin G (IgG) antibodies. Upper: agarose gel electrophoresis of RIP–quantitative polymerase chain reaction (qPCR) products. Lower: relative enrichment of LNPPS in PDCD5 by qRT‐PCR. (E) RNA fluorescence in situ hybridisation (FISH) and immunofluorescence (IF) assays showing the co‐localisation of LNPPS (Cy3‐labelled, red) and PDCD5 (Alexa488‐labelled, green) in 5637 and J82 cells. Blue: 4′,6‐diamidino‐2‐phenylindole (DAPI). (F) Western blotting of PDCD5 in samples pulled down by full‐length (FL) or truncated LNPPS1 (F1: 1–306 nt, F2: 121–360 nt, F3: 251–424 nt). (G) RNA pull‐down assays showing the interaction between a series of truncated LNPPS and Flag‐PDCD5 in HEK293T cells. LNPPS^FL^: the FL of LNPPS; LNPPS^121‐251^ ^nt^: the 121–251 nt of LNPPS; LNPPS^Δ121‐251^ ^nt^: the deletion mutant of 121–251 nt for LNPPS. (H) Left: putative interaction between PDCD5 and LNPPS predicted by catPAPID database. Schematic diagrams of PDCD5 truncations are shown at the bottom. Δ1–30, Δ31–60, Δ61–90 and Δ91–125 refer to protein fragments with deletion of regions 1–30, 31–60, 61–90 and 91–125, respectively. Right: relative enrichment of LNPPS in truncated PDCD5 tested by RIP–qPCR. Not significant (n.s.): >.05, ^*^ *p* < .05, ^**^ *p* < .01 and ^***^ *p* < .001

Interaction with specific protein(s) is one of the most important mechanisms by which lncRNAs exert their functions.^21^ Since LNPPS is mainly located in the nucleus, RNA pull‐down assays were followed by MS to identify the possible LNPPS–protein(s) complex. The MS data are shown in Table S5. We first focused on 103 specific potential proteins (≤25 kD) since this area showed more distinctly differential bands in LNPPS sense‐probe pull‐down samples compared with antisense control in BC cells (Figures 4A and S3A). To screen out the more credible proteins for further validation, these 103 proteins were sorted and filtered by three MS‐related indicators (Figure 4B). Following the above analysis pipeline, three candidate proteins were identified and then validated by RNA pull‐down assays. The results showed that only PDCD5 specifically bound to LNPPS (Figures 4C and S3B). RIP assays verified the interaction between PDCD5 and LNPPS in 5637 and J82 cells (Figure 4D). RNA FISH and IF assays indicated that endogenous LNPPS was mainly co‐localised with PDCD5 in the nucleus (Figure 4E).

To further determine the regions responsible for the interaction between LNPPS and PDCD5, we constructed three truncated fragments of LNPPS (F1: 1–306 nt; F2: 121–360 nt; F3: 251–424 nt) and used them in RNA pull‐down assays. The results showed that F1 and F2, but not F3, could bind to PDCD5, suggesting that the region of 121–251 nt in LNPPS was required for the interaction with PDCD5 (Figure 4F), which was further validated by using the internal region (121–251 nt) of LNPPS (Figure 4G). By using catRAPID algorithm for estimating the binding propensity of protein/RNA pairs, a.a. 1–90 domain of PDCD5 showed a stronger binding strength with LNPPS. Therefore, PDCD5 was divided into four fragments, as shown in the left panel of Figure 4H. The RIP assays revealed that deletion of the domain (a.a. 1–30) almost abolished the binding of PDCD5 and LNPPS (Figure 4H, right panel). These results propose that LNPPS and PDCD5 form the RNA–protein complex specifically through the region (121–251 nt) of LNPPS and the N‐terminal domain (a.a. 1–30) of PDCD5.

### 3.4 LNPPS protects PDCD5 from ubiquitin‐mediated protein degradation by masking its K20 site ubiquitination

**FIGURE 5:**
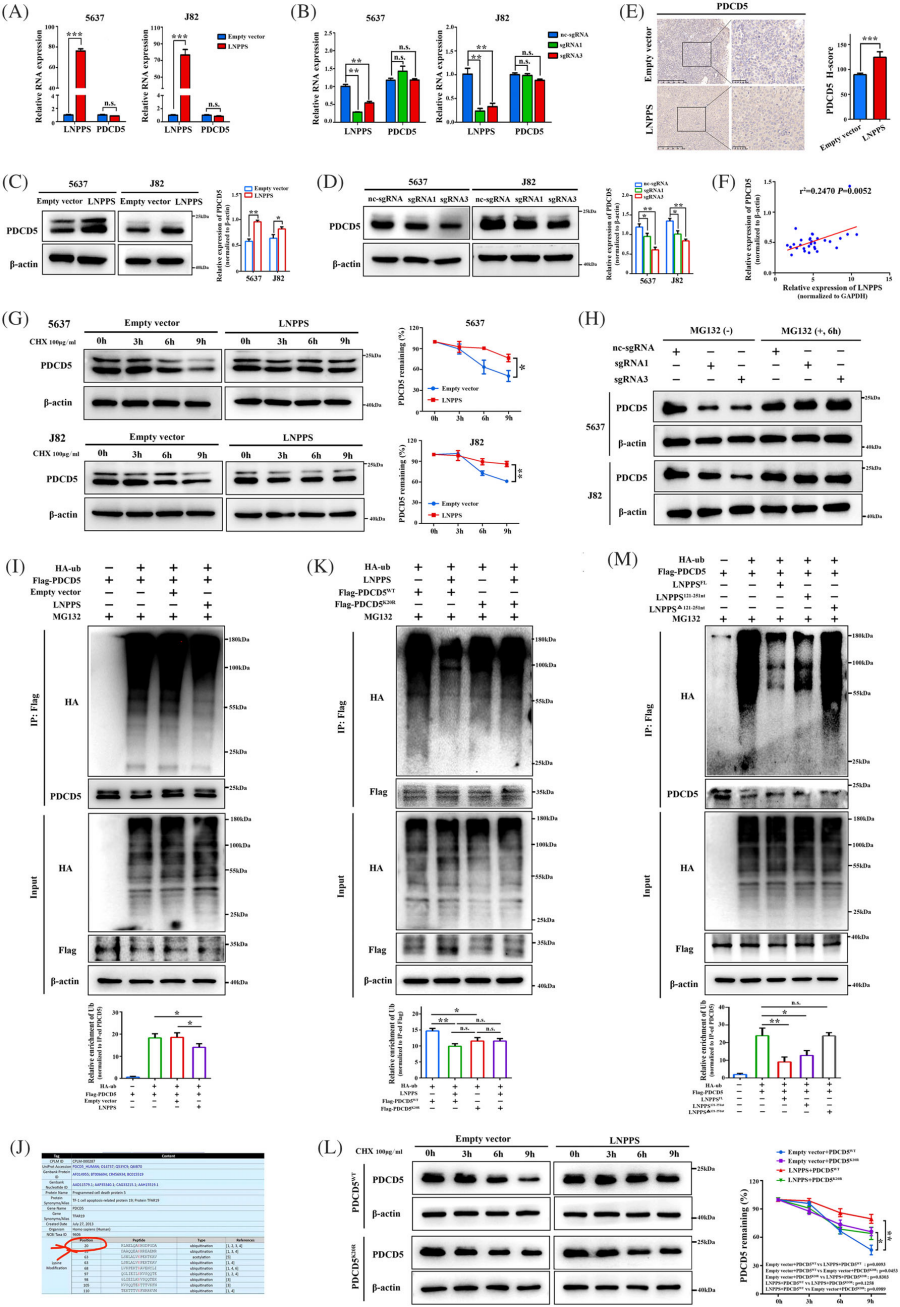
LNPPS protects programmed cell death 5 (PDCD5) from protein degradation by masking its K20 site ubiquitination. (A and B) The mRNA levels of PDCD5 after LNPPS overexpressed (A) and knockdown (B) in 5637 and J82 cells by quantitative reverse transcription‐polymerase chain reaction (qRT‐PCR). (C and D) The protein levels of PDCD5 after LNPPS overexpressed (C) and knockdown (D) in 5637 and J82 cells by Western blotting analysis. (E) Representative images of immunohistochemistry (IHC) staining of PDCD5 in paraffin‐embedded xenograft tumours from LNPPS stably overexpressed mice models. Scale bars: 250 and 50 μm, respectively. (F) The correlation between the expression of LNPPS and PDCD5 in bladder cancer (BC) tissues. The levels of LNPPS and PDCD5 were examined in tumour tissues from 30 BC patients by qRT‐PCR and Western blotting analysis, respectively. (G) Left: the PDCD5 protein levels in cells expressing LNPPS or empty vector and then treated with cycloheximide (CHX) (100 μg/ml). Right: quantisation of PDCD5 degradation rate by grey scale analysis. (H) The PDCD5 protein levels in LNPPS‐silencing cells or control cells and then treated with MG132 (10 μM, 6 h). (I) The effect of LNPPS on exogenous PDCD5 ubiquitination. HEK293T cells were co‐transfected with indicated sets of plasmids and treated with MG132. Cell lysates were immunoprecipitated with anti‐Flag antibody followed by immunoblotting (IB) assays. (J) The predicted ubiquitination sites of PDCD5 by CPLM database. (K) Co‐immunoprecipitation (Co‐IP) assays showing the role of PDCD5–K20 site in the LNPPS‐regulated ubiquitination of exogenous PDCD5 in HEK293T. (L) The half‐life of PDCD5^WT^ and PDCD5^K20R^ with or without LNPPS overexpression under CHX treatment. (M) Co‐IP assays showing the effect of the full‐length LNNPS or a series of truncated LNPPS on the ubiquitination of PDCD5. Not significant (n.s.) >.05, ^*^ *p* < .05, ^**^ *p* < .01 and ^***^ *p* < .001

Next, we sought to elucidate the relative contribution of the specific interaction between LNPPS and PDCD5. qRT‐PCR showed that the RNA levels of PDCD5 remained steady after either overexpression or knockdown of LNPPS in BC cells (Figure 5A,B). Importantly, the protein levels of PDCD5 were significantly upregulated upon LNPPS overexpression, whereas they were decreased with silencing of LNPPS in 5637 and J82 cells (Figure 5C,D). Similar results were observed in subcutaneous tumours overexpressing LNPPS (Figure 5E). Moreover, we found a significantly positive correlation between the expression levels of PDCD5 and LNPPS in tumour tissues from 30 BC patients (Figure 5F). These findings suggest that post‐translational regulation may be responsible for the LNPPS‐induced PDCD5 dysregulation.

To explore the dysregulation mechanism of PDCD5, LNPPS‐overexpressing cells were treated with cycloheximide (CHX) (a de novo protein synthesis inhibitor). The half‐life of PDCD5 protein was remarkably prolonged in the LNPPS‐overexpressing cells than in the control group (Figure 5G). Notably, the suppressive effect of silencing LNPPS on PDCD5 protein was offset by the proteasome inhibitor MG132 (Figure 5H). To further investigate whether the increased PDCD5 abundance induced by LNPPS is involved in ubiquitin–proteasome degradation, ubiquitination IP assays were performed, and the results showed that the levels of exogenous and endogenous PDCD5 ubiquitination were inhibited when LNPPS was overexpressed (Figures 5I and S3C). Moreover, the CPLM database indicated that there was one ubiquitinated lysine (K) residue (K20) in the N‐terminal domain (a.a. 1–30) of PDCD5 (Figure 5J). As expected, the K20 functioned as a PDCD5 ubiquitination acceptor site, whose mutation decreased the ubiquitination level of PDCD5 (Figures 5K and S3D). There was no significant difference between the inhibitory effect of LNPPS on PDCD5 ubiquitination and the reduction of ubiquitination brought on by the PDCD5–K20 site mutation. Consistently, the protein stability assays also supported that the stabilisation effect of LNPPS on PDCD5 might occur via the K20 ubiquitination site (Figure 5L). Thus, we attempted to investigate whether the stabilisation effect of LNPPS on PDCD5 was due to the interaction between them, which masked its K20 site ubiquitination. Ubiquitination IP assays showed that the full‐length and 121–251 nt LNPPS, but not the deletion mutant of 121–251 nt, could inhibit PDCD5 ubiquitination and increase the levels of PDCD5 protein (Figures 5M and S3E,F). On the other hand, we generated a specific mutant of PDCD5 (PDCD5^K20^) that mutated the 1–30 a.a. region of PDCD5 but preserved its K20 site. RIP assays indicated that the mutant PDCD5^K20^ lacked the interaction with LNPPS (Figure S3G). Ubiquitination IP assays further demonstrated that LNPPS reduced the ubiquitination level of WT PDCD5, but did not affect the ubiquitination of mutant PDCD5^K20^ (Figure S3H). These findings suggest that LNPPS suppresses the degradation of PDCD5 by masking its K20 site ubiquitination.

### 3.5 LNPPS activates p53 signalling in a PDCD5‐dependent manner

**FIGURE 6:**
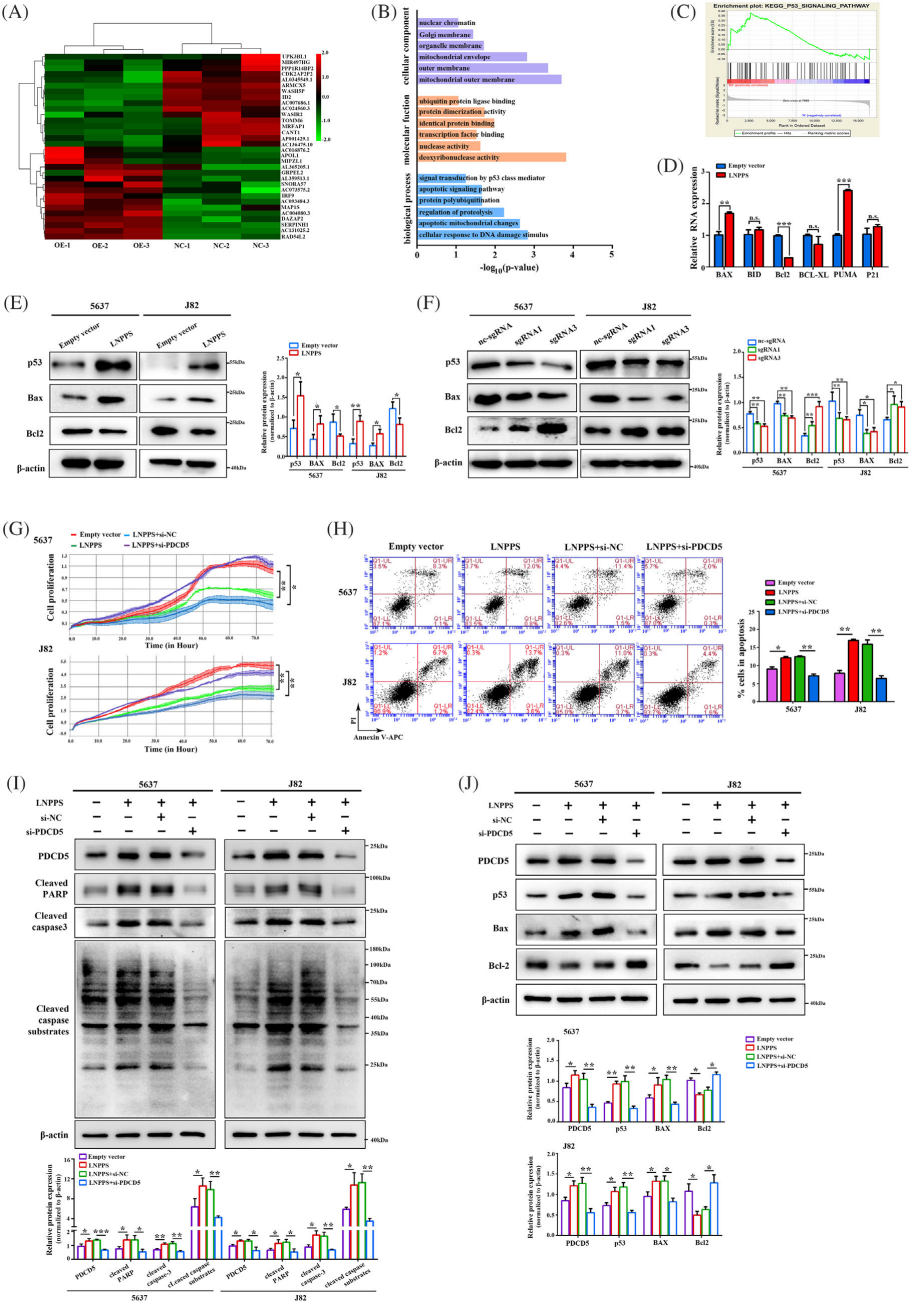
LNPPS enhances p53 signalling in a programmed cell death 5 (PDCD5)‐dependent manner. (A) Heatmap of differently expressed genes in LNPPS stable overexpressed 5637 cells compared with control group. Red and green colours indicated high and low expression levels relative to global median, respectively. (B) Gene Ontology (GO) analysis of differently expressed genes in LNPPS stable overexpressed 5637 cells compared with control group. (C) Gene set enrichment analysis (GSEA) analysis showing the activation of p53 signalling pathway in LNPPS stable overexpressed cells. (D) The RNA levels of several p53 targets after LNPPS overexpressed in 5637 cells. (E and F) The protein levels of p53 and its several targets after LNPPS overexpression (E) or knockdown (F) in cells. (G and H) The cell growth dynamics (G) and cell apoptosis assays (H) of 5637 and J82 cells expressing LNPPS or empty vector plasmid with or without si‐PDCD5 by xCELLigence system and flow cytometry. (I) Western blotting showing the role of PDCD5 in LNPPS‐regulated cell apoptosis. (J) The protein levels of p53 (generic) and its targets after co‐transfected with LNPPS‐overexpression plasmid and specific siRNA for PDCD5 in cells. Not significant (n.s.) >.05, ^*^ *p* < .05, ^**^ *p* < .01 and ^***^ *p* < .001

To provide insights into the mechanisms by which LNPPS regulated BC development, RNA‐seq was used to examine the gene expression profiles in LNPPS‐overexpressing cells (raw data accessible via GSE190917). Eighty‐two genes were upregulated and 102 genes were negatively related to LNPPS overexpression (Figure 6A and Table S6). According to the GO and KEGG pathway enrichment analyses, the LNPPS‐dependent transcriptions in BC were enriched in the apoptotic signalling pathway, signal transduction by p53 class mediator and protein polyubiquitination (Figures 6B and S4A). GSEA also revealed that the gene set of the p53 signalling pathway was positively correlated with LNPPS overexpression in BC cells (Figure 6C). Fischer^22^ compiled an updated list of p53 target genes from high‐throughput studies and individual gene analyses. We examined the p53 potential targets in LNPPS‐overexpressing cells based on Fischer's list. It turned out that 15 genes were overlapped and most of them were involved in p53‐mediated apoptosis, such as BAX, BBC3 (PUMA), PMAIP1, PIDD1, TP53AIP1, etc. (Figure S4B). qRT‐PCR and WB analysis indicated that the levels of p53 and its pro‐apoptotic target genes, BAX and PUMA, were increased by LNPPS overexpression, while the anti‐apoptotic Bcl2 was decreased. The opposite effect was observed in the LNPPS‐knockdown cells (Figures 6D–F and S4C,D). We further found that inhibition of p53 abrogated the ability of LNPPS to trigger cell apoptosis (Figure S4E). Notably, 5637 and J82 cells harbour TP53 missense mutations at non‐hotspot codons in the DNA‐binding domain (DBD).^23^ Some high‐throughput screens have reported that there is considerable heterogeneity in the degree of residual DNA‐binding activity and dysfunction between different missense mutations in p53 DBD.^24^, ^25^ Considering LNPPS still induces the p53 signalling pathway in 5637 and J82 cells, we asked whether the mutant p53 has some residual transcriptional activity and function in the two kinds of BC cells. Dual‐luciferase reporter assays showed that mutant p53 had a certain residual DNA‐binding ability to WT p53 response elements (RE) compared to mutant WT‐p53RE and non‐p53RE (empty vector) in 5637 and J82 cells (Figure S4F). ChIP–qPCR assays revealed that the mutant p53 in 5637 and J82 cells had slight activities for binding sequences in the promoters of several WT p53 targets such as BAX and PUMA, but these activities were lower than those of WT p53 in RT4 cells (Figure S4G). Surprisingly, we observed that LNPPS increased the residual transcriptional activity of mutant p53 to a certain extent in 5637 and J82 cells (Figure S4F,H), which might be the basis for LNPPS exerting its tumour suppressor effect through the p53 pathway.

Next, we assessed whether the repressor role of LNPPS was dependent on PDCD5. Silencing of PDCD5 partly reversed the suppressed effect of LNPPS overexpression on BC cell viability (Figure 6G). PDCD5 silencing also abrogated the ability of LNPPS to trigger cell apoptosis (Figure 6H,I). Moreover, we observed that the dysregulation of p53 and its downstream targets caused by LNPPS was rescued by PDCD5 knockdown (Figure 6J). These results suggest that LNPPS functions as a tumour suppressor in a PDCD5‐dependent manner, which is related to activating p53 signalling.

### 3.6 LNPPS serves as a bridge to connect PDCD5 with p53 and maintains p53 accumulation by means of PDCD5

**FIGURE 7:**
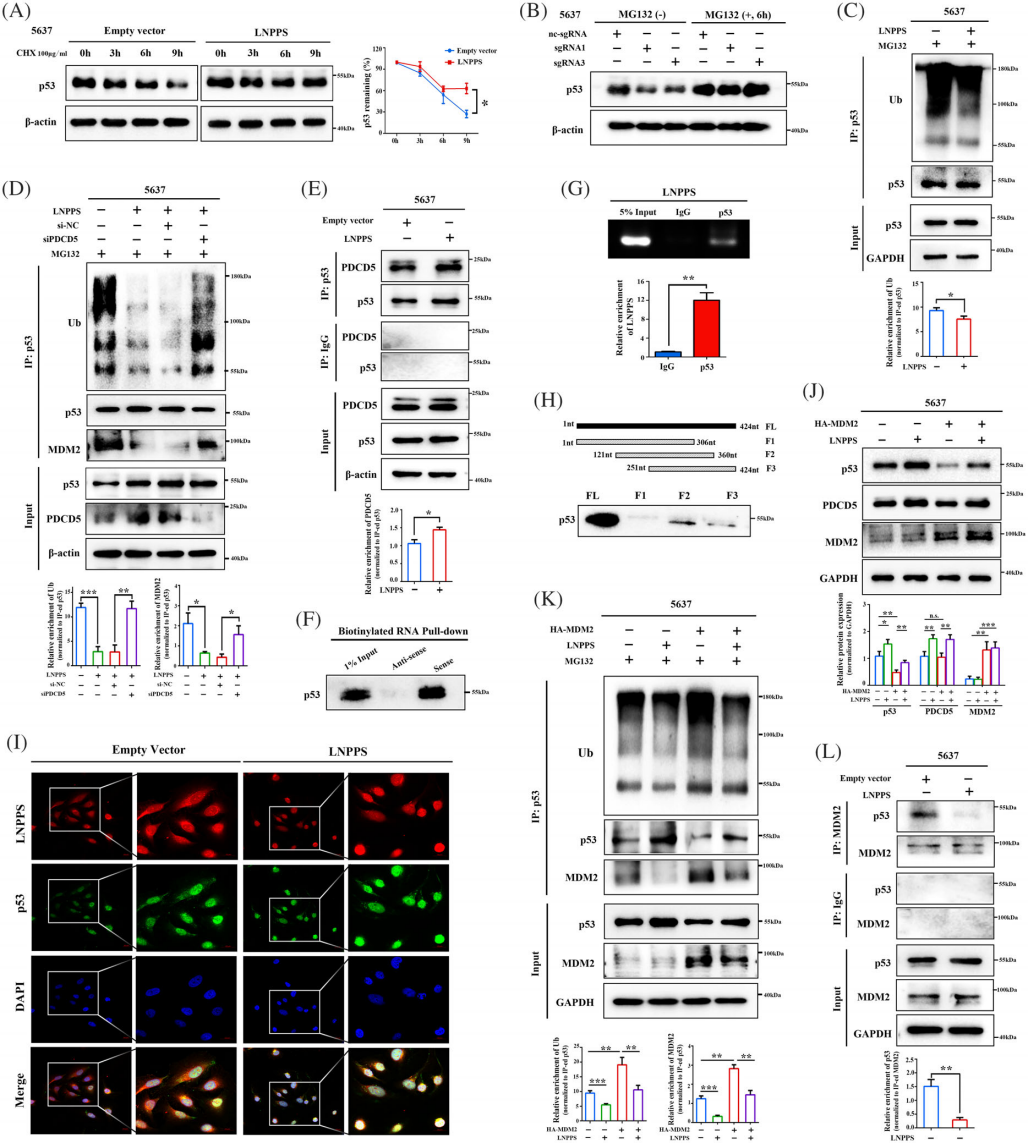
LNPPS blocks mouse double minute 2 homologue (MDM2)‐mediated p53 ubiquitination and degradation by means of programmed cell death 5 (PDCD5). (A) The p53 protein levels in 5637 cells expressing LNPPS or empty vector under cycloheximide (CHX) treatment. (B) Western blotting analysis showing the p53 levels in 5637 cells transfected with sgRNAs specific for LNPPS or nc‐sgRNA and then treated with MG132. (C) The effect of LNPPS on endogenous p53 ubiquitination. 5637 cells were co‐transfected with indicated sets of plasmids and treated with MG132. Cell lysates were immunoprecipitated with anti‐p53 antibody followed by immunoblotting (IB) assays. (D) The role of PDCD5 in the LNPPS‐regulated endogenous p53 ubiquitination and the MDM2–p53 interaction in 5637 cells. (E) Co‐immunoprecipitation (Co‐IP) assays showing the effect of LNPPS on the interaction between p53 and PDCD5 in 5637 cells. (F) Western blotting analysis after RNA pull‐down assays showing the binding of LNPPS and p53 in 5637 cells. (G) RNA immunoprecipitation (RIP) assays with anti‐p53 and anti‐immunoglobulin G (IgG) antibodies in 5637 cells. Upper: agarose gel electrophoresis of RIP–qPCR products. Lower: relative enrichment of LNPPS in p53 by quantitative reverse transcription‐polymerase chain reaction (qRT‐PCR). (H) Western blotting analysis of PDCD5 in samples pulled down by full‐length (FL) or truncated LNPPS (F1: 1–306 nt, F2: 121–360 nt, F3: 251–424 nt). (I) Subcellular distribution of p53 and co‐localisation between p53 and LNPPS in LNPPS‐overexpressed J82 cells. Red: Cy3‐labelled LNPPS; green: Alexa488‐labelled p53; blue: 4′,6‐diamidino‐2‐phenylindole (DAPI). Scale bars: 20 μm. (J) The levels of p53 and PDCD5 in 5637 cells after expressed indicated sets of plasmids. (K) The role of LNPPS in MDM2‐mediated endogenous p53 ubiquitination in 5637 cells. (L) The effect of LNPPS on the interaction between p53 and MDM2. Not significant (n.s.) >.05, ^*^ *p* < .05, ^**^ *p* < .01 and ^***^ *p* < .001

As mentioned above, LNPPS not only activated p53 signalling but also participated in regulation of p53 itself. We found that overexpression and silencing of LNPPS had negligible effects on the RNA level of p53, suggesting that the promoting effect of LNPPS on p53 might not be involved in transcriptional regulation (Figure S5A,B). IHC assays showed that p53 was increased in the subcutaneous tumours overexpressing LNPPS compared to the control group (Figure S5C). The expression levels between LNPPS and p53 showed a positive correlation in 30 BC tissues (Figure S5D). Next, upon exposure to CHX, LNPPS prolonged the half‐life of p53 protein (Figures 7A and S5E). On the other hand, the protein levels of p53 were significantly decreased once LNPPS was knocked down, whereas the reduction of p53 caused by LNPPS knockdown was almost blocked by MG132 treatment (Figures 7B and S5F). These data indicate that LNPPS increases p53 protein expression in BC cells by suppressing its protein degradation.

It is known that p53 protein is mainly degraded via the ubiquitin–proteasome pathway.^26^ Indeed, IP assays showed that the ubiquitination of endogenous p53 protein was diminished when LNPPS was ectopically expressed (Figures 7C and S5G). Since LNPPS was found to activate p53 signalling in a PDCD5‐dependent manner, we subsequently investigated whether the inhibited p53 ubiquitination by LNPPS also relied on PDCD5. PDCD5 knockdown partly reversed the inhibition of endogenous ubiquitinated p53 levels in LNPPS‐overexpressing cells (Figures 7D and S5H). A previous study reported that PDCD5 physically interacts with p53.^18^ To investigate whether LNPPS affects p53 ubiquitination by regulating the PDCD5–p53 interaction, co‐immunoprecipitation (Co‐IP) assays were carried out, and the results demonstrated that overexpression of LNPPS resulted in an increased interaction between PDCD5 and p53 (Figures 7E and S5I). In parallel, knockdown of LNPPS disrupted the binding of PDCD5 and p53 (Figure S5J). Meanwhile, we observed the co‐localisation between PDCD5 and p53, particularly in LNPPS‐overexpressing BC cells (Figure S5K). In addition, there was a positive correlation between the expression levels of PDCD5 and p53 in BC patients (Figure S5L). These findings imply that LNPPS mediates the PDCD5–p53 interaction and prevents it from degradation through the ubiquitin–proteasome pathway.

Strikingly, we observed that LNPPS was similarly the RNA‐binding partner of p53, as it physically interacted with p53 in BC cells (Figure 7F,G). RNA FISH assays followed by IF staining of p53 supported the association between LNPPS and p53, which were mainly localised in the nucleus of 5637 and J82 cells (Figures 7I and S6C). RNA pull‐down assays with different biotin‐labelled truncations of LNPPS further indicated that F2 showed the strongest pull‐down effect, followed by F3 and F1, suggesting that the common part of all three truncations (251‒306 nt) might contribute most to the binding of LNPPS to p53 (Figure 7H). To dissect whether LNPPS functions as a bridge to mediate the interaction between PDCD5 and p53, RNA pull‐down assays without PDCD5 were performed, and demonstrated that silencing of PDCD5 had little effect on the binding of p53 to LNPPS (Figure S5M). In addition, IF assays and WB analysis indicated that cytoplasmic p53 and PDCD5 were downregulated upon LNPPS overexpression, while their expressions in the nucleus were correspondingly increased, suggesting that LNPPS promoted the nuclear translocation of p53 and PDCD5 in BC cells (Figures 7I and S6). Considering that protein proteasomal degradation primarily takes place in the cytoplasm, the enhanced nuclear entry of the LNPPS/p53/PDCD5 complex might exert a synergistic effect on prevention of protein degradation. Altogether, these data indicate that LNPPS functions as a bridge to connect PDCD5 with p53 and prevents p53 from ubiquitination degradation, providing a basis for the dysregulation of p53 in BC development.

### 3.7 LNPPS blocks MDM2‐mediated p53 ubiquitination and degradation

MDM2, an E3 ubiquitin–protein ligase, binds to p53 and facilitates its polyubiquitination with subsequent 26S proteasome degradation.^27^ Since we revealed the inhibitory effect of LNPPS on p53 ubiquitination, we next assessed whether LNPPS was involved in MDM2‐mediated p53 ubiquitination and degradation in BC cells. Indeed, we found that MDM2 overexpression increased the endogenously ubiquitinated p53 levels, leading to the reduction of p53 protein in 5637 and J82 cells (Figures 7J,K and S7A,B). Importantly, MDM2‐mediated p53 ubiquitination and degradation were, at least partially, impaired by LNPPS (Figures 7K and S7B). Next, we investigated how LNPPS suppressed MDM2‐mediated p53 ubiquitination. Co‐IP assays showed that the binding of MDM2 and p53 was much weaker when LNPPS was overexpressed, regardless of whether anti‐MDM2 antibody or anti‐p53 antibody was used as bait (Figures 7L and S7C). Similar results were also observed in J82 cells (Figure S7D). Furthermore, the weaker strength of interaction could be reversed by silencing PDCD5, suggesting that LNPPS suppressed the binding of MDM2 and p53 partially dependent on PDCD5 (Figures 7D and S5H). Of note, we observed that MDM2 had little effect on PDCD5 proteins (Figures 7J and S7A). MDM2 also did not bind to PDCD5 directly, ruling out the possibility that MDM2 contributed to the dysregulation of PDCD5 (Figure S7E,F). Collectively, our results suggest that LNPPS mediates the binding of PDCD5 to p53, which disrupts the p53/MDM2 complex, resulting in increased p53 accumulation by suppressing MDM2‐mediated p53 ubiquitination and degradation.

### 3.8 m^6^A modification is involved in the downregulation of LNPPS in BC cells

**FIGURE 8:**
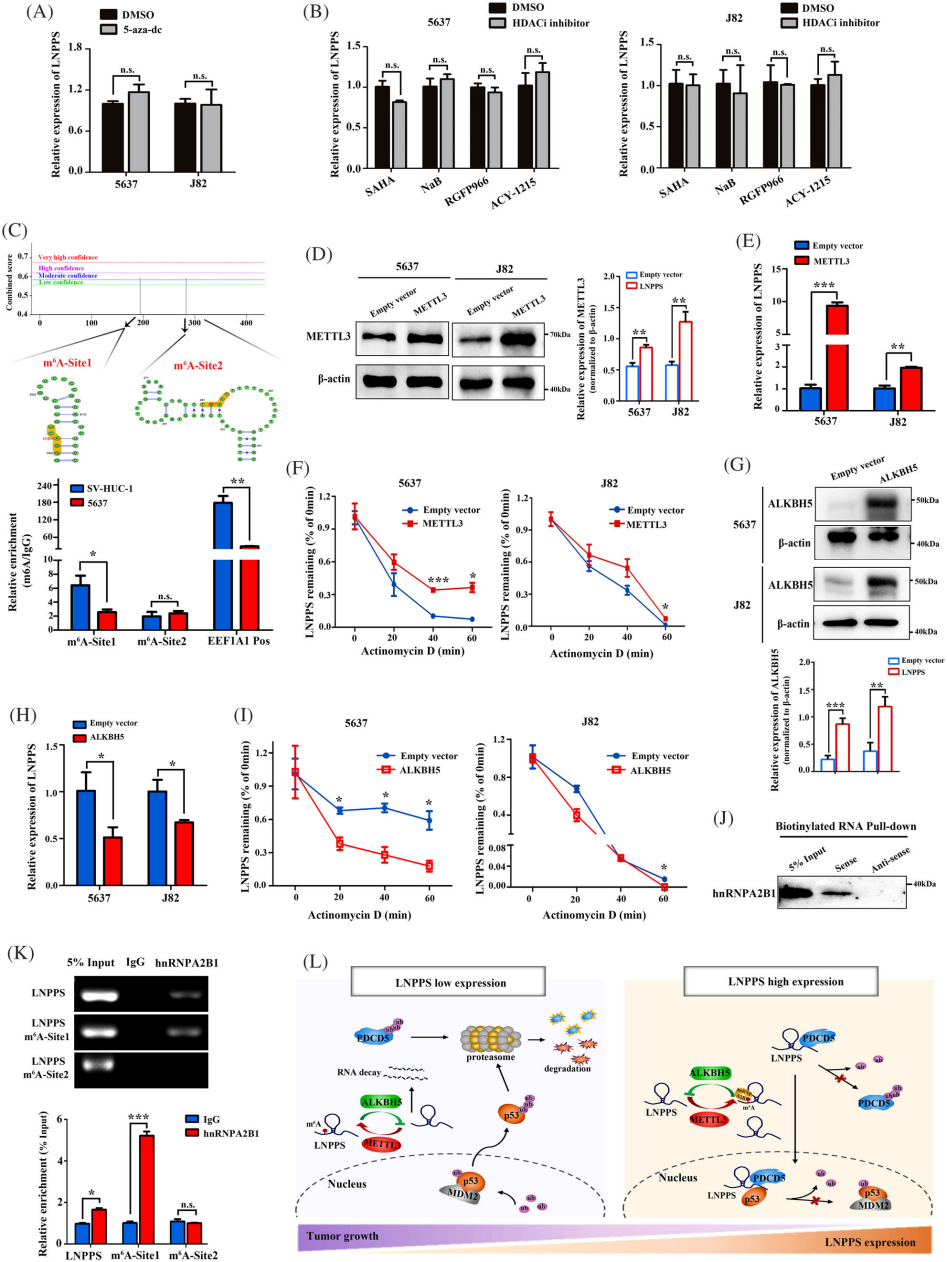
N6‐methyladenosine (m^6^A) modification is involved in the downregulation of LNPPS in bladder cancer (BC) cells. (A) The expression of LNPPS after treatment with 5‐aza‐dc (5 μM) or dimethyl sulfoxide (DMSO) for 4 days in 5637 and J82 cells. (B) The expression of LNPPS under treatment with SAHA (2 μM), NaB (2 μM), RFGP966 (1 μM) and ACY‐1215 (4 μM) for 24 h in BC cells. (C) m^6^A methylated RNA immunoprecipitation (MeRIP) assays showing the enrichment of m^6^A in SV‐HUC‐1 and 5637 cells. Upper: the predicted m^6^A sites in LNPPS by SRAMP. Lower: quantitative reverse transcription‐polymerase chain reaction (qRT‐PCR) results of MeRIP assays. EEF1A1 Pos: the positive control. (D and G) The overexpression efficiency of methyltransferase‐like 3 (METTL3) (D) and AlkB homologue 5 (ALKBH5) (G) after transfected with its overexpression plasmid in BC cells. (E and H) The expression of LNPPS after overexpressed METTL3 (E) and ALKBH5 (H) by qRT‐PCR. (F and I) The decay rates of LNPPS under treatment with 10 μM actinomycin D for indicated times in METTL3 overexpressing cells (F) or ALKBH5 overexpressing cells (I). (J) Western blotting analysis after RNA pull‐down assays showing the binding of LNPPS and hnRNPA2B1 in 5637 cells. (K) RNA immunoprecipitation (RIP) assays with anti‐hnRNPA2B1 and anti‐immunoglobulin G (IgG) antibodies in 5637 cells. Upper: agarose gel electrophoresis of RIP–qPCR products. Lower: relative enrichment of LNPPS and its m^6^A sites in hnRNPA2B1 by qRT‐PCR. (L) The schematic model for the mechanisms of LNPPS in BC tumourigenesis. ^*^ *p* < .05, ^**^ *p* < .01 and ^***^ *p* < .001

To study the mechanisms by which LNPPS is downregulated in BC, we first treated BC cells with 5‐aza‐dC. qRT‐PCR showed that the DNA methyltransferase inhibitor had no significant effect on LNPPS expression (Figures 8A and S8A). The contribution of histone acetylation to LNPPS expression was then tested by treatment with broad‐spectrum or specific histone deacetylase (HDAC) inhibitors. The data indicated that SAHA, NaB and specific inhibitors of HDAC3, 6 also hardly affected LNPPS expression (Figures 8B and S8B,C). Therefore, the downregulation of LNPPS in BC cells might not be related to DNA methylation and histone acetylation.

The m^6^A modification plays a crucial role in RNA metabolism, including its transcription splicing, subcellular localisation, translation and decay.^28^ We wondered whether m^6^A was responsible for the downregulation of LNPPS in BC. According to the results from a public prediction server SRAMP database, we found two RRACU m^6^A sequence motifs in LNPPS (site 1, ch5: 149 428 096; site 2, ch5: 149 428 007). MeRIP assays verified that the enrichment of m^6^A by LNPPS at site 1 was lower in 5637 cells than in SV‐HUC‐1 cells (Figure 8C). As a dynamic and reversible modification, the state of m^6^A is determined by m^6^A methyltransferases (‘writers’) and demethylases (‘erasers’). METTL3 and METTL14 have been identified as the key ‘writers’, while ALKBH5 and FTO are the main ‘erasers’.^29^ We then evaluated the correlation between these m^6^A regulators and LNPPS in BC tissues. qRT‐PCR indicated that the level of METTL3 was positively correlated with the expression of LNPPS in BC tissues and that ALKBH5 was negatively correlated with LNPPS, whereas METTL14 and FTO had no significant correlation with LNPPS (Figure S8D–G). Next, we upregulated these m^6^A ‘writers’ and ‘erasers’ with their overexpression plasmids in BC cells (Figures 8D,G and S8H,J). Overexpression of METTL3, rather than METTL14, significantly increased the expression of LNPPS (Figures 8E and S8I). Similarly, the downregulation of LNPPS was observed only when ALKBH5 was overexpressed, instead of FTO (Figures 8H and S8K). These findings suggest that m^6^A modulates the expression of LNPPS in BC cells.

We then investigated the mechanisms by which m^6^A regulated LNPPS expression in BC cells. By using the actinomycin D treatments to intercept the RNA synthesis, we found that METTL3 overexpression resulted in a prolonged half‐life of LNPPS (Figure 8F). Conversely, the half‐life of LNPPS was decreased in ALKBH5‐overexpressing BC cells (Figure 8I). The data suggest that METTL3‐ and ALKBH5‐mediated m^6^A modifications can specifically regulate LNPPS stability, providing a basis for its dysregulation in BC. According to our previous MS results of RNA pull‐down assays, we found that heterogeneous nuclear ribonucleoprotein A2B1 (hnRNPA2B1, working as m^6^A reader) might be the potential LNPPS‐binding protein (Table S5). RNA pull‐down assays revealed that hnRNPA2B1 was enriched by LNPPS sense‐probe rather than antisense control (Figure 8J). To further explore the essential role of hnRNPA2B1 in LNPPS methylation, RIP assays were carried out and demonstrated that m^6^A site 1 of LNPPS was the binding target of hnRNPA2B1, which was consistent with our MeRIP data (Figure 8K). Overall, these findings indicate that hnRNPA2B1 binds to the m^6^A‐bearing LNPPS and may serve as an intermediary for LNPPS stability mediated by METTL3 and ALKBH5. A schematic model depicting the epigenetic modification and regulatory network of LNPPS is shown in Figure 8L.

## 4 DISCUSSION

In this study, we verified that LNPPS, a newly characterised lncRNA, was downregulated due to low m^6^A modification in BC, and it could suppress the viability of BC cells by activating p53‐related apoptosis. Mechanistically, LNPPS functioned as a bridge to link PDCD5 and p53, competitively impairing MDM2‐mediated p53 ubiquitination. Meanwhile, the increasing interaction between LNPPS and PDCD5 upregulated PDCD5 by blocking its degradation through masking its K20 site ubiquitination. Therefore, the present study highlights the suppressive role of the LNPPS/PDCD5/p53/MDM2 regulatory axis in BC and provides promising therapeutic targets for BC patients.

The recent revolution in genome and transcriptome sequencing has led to the discovery of many novel lncRNAs. However, the potential involvement of lncRNAs in BC remains enigmatic. Through a combination of transcriptomic and bioinformatic analyses, we found that a novel lncRNA transcript ENST00000622374 (LNPPS), located on the reverse strand of chromosome 5q32, was significantly downregulated in BC specimens. The 5q32 locus encodes multiple non‐coding RNAs. Among them, lncRNA CARMN, miR‐143 and miR‐145 are closest to LNPPS, which are localised in the forward strand of 5q32. Several studies have reported that all three non‐coding transcripts can accelerate the development of atherosclerosis.^30^ Moreover, CARMN was found to control breast cancer stem cell self‐renewal by regulating wnt10a via the formation of functional triplex.^31^ Nevertheless, the function and mechanisms of LNPPS in BC are far from being identified. Here, we reported a suppressive effect of LNPPS on BC progression in vitro and in vivo. More interestingly, LNPPS functioned as the molecular scaffold to connect PDCD5 with p53, which facilitated the PDCD5/p53‐associated apoptosis.

PDCD5, a highly conserved protein, was first identified as an apoptosis‐promoting signal in many diseases, including cancers.^32^ When cells undergo apoptosis, PDCD5 is rapidly upregulated and translocated from the cytoplasm to the nucleus.^33^ Multiple recent studies have attempted to explore the underlying mechanisms of PDCD5 upregulation in promoting apoptosis. Kwak et al.^34^ found that STK31 interacts with PDCD5 and sustains its stability. Park et al.^35^ further revealed that deubiquitinase OTUD5 directly binds to PDCD5 and promotes its accumulation by mediating PDCD5 deubiquitination at Lys‐97/98 upon genotoxic stress‐induced apoptosis. Although previous studies have investigated the association between PDCD5 and multiple cellular proteins, the roles of lncRNAs in this process have remained largely unexplored. In this study, we found that lncRNA LNPPS, which served as a novel PDCD5‐interacting partner, bound the N‐terminal domain of PDCD5 (a.a. 1–30) through its internal region (121–251 nt), therefore protecting PDCD5 from ubiquitin–proteasome degradation by masking its K20 site ubiquitination. Furthermore, the increased nuclear translocation of PDCD5 caused by LNPPS might be another potential way to prevent PDCD5 degradation in the cytoplasm. Our findings not only indicate that lncRNAs took part in the accumulation of PDCD5 but also provide interesting insights into the relationship between lncRNAs and post‐translational modification of PDCD5. Notably, our experimental data showed that the ubiquitination inhibition and protein stabilisation of WT PDCD5 by LNPPS were slightly stronger than the effect caused by the PDCD5–K20 site targeted mutation, although the difference was not statistically significant. Possible explanations for this include that the targeted mutation of K20, while blocking PDCD5 ubiquitination at this site, may pose the risk of partially altering the balance of the PDCD5 ubiquitination network to some extent. Additionally, it is also possible that LNPPS may influence PDCD5 ubiquitination through additional mechanisms, which requires investigation in further research. Furthermore, one limitation to this study is that we are not sure which kinds of roles are played by E3 ubiquitin ligases in the LNPPS‐mediated regulatory network for PDCD5 ubiquitination and degradation, which deserves further investigation in subsequent studies.

As a known master tumour suppressor, p53 regulates a variety of cellular processes, notably cell cycle arrest and apoptosis.^36^ However, mutations in p53 are frequently found in BC, which result in varying degrees of tumour‐suppressive dysfunction, and some mutants even gain novel functions that are necessary for tumourigenesis.^37^ The BC cell lines used in this study, 5637 and J82, both harbour TP53 missense mutations at non‐hotspot codons in the p53 [5637, codon 280 (Arg > Thr); J82, codon 271 (Glu > Lys), codon 274 (Val > Phe) and codon 320 (Lys > Asn)].^23^ A high‐resolution missense mutation analysis has reported that there are at least three mutant p53 subtypes: mutants with no activity, mutants with reduced but residual activity and mutants with activity comparable to that of WT p53.^24^ Intriguingly, our observations showed that the mutant p53 had slight residual DNA‐binding activity in 5637 and J82 cells compared to that of WT p53 in RT4 cells. Furthermore, LNPPS enhanced the residual ability of mutant p53 to transactivate several pro‐apoptotic targets in 5637 and J82 cells. On the other hand, we noticed that LNPPS also had a pro‐apoptotic effect in RT4 cells (harbour WT p53) and similarly increased the expression of WT p53 proteins. Additionally, LNPPS promoted the p53 signalling pathway and inhibited the ubiquitination of WT p53 in a PDCD5‐dependent manner (Figure S9). These findings, from a side‐by‐side view, suggest that the suppressive effect of LNPPS in 5637 and J82 cells is most likely based on the residual WT activity of mutant p53. The possible mechanism includes that increased accumulation of p53 mutants by LNPPS partially compensates for the qualitative defects, which resembles the mass effect: the more proteins are available, the higher the probability they are to bind DNA, and the more similar they are to WT p53. Currently, the interrelation among p53 tumour‐derived mutations and function has remained an open question of great interest. This study investigated the promoting effect of LNPPS on mutant p53 with residual transcriptional activity, hoping to provide insights into the complex mutation–function network of p53.

It is well known that PDCD5 functions as a p53‐positive regulator by blocking the interaction of p53 with E3 ubiquitin ligase MDM2.^17^ Importantly, we found that LNPPS remarkably enhanced the protective effect of PDCD5 on the protein stability of p53. In brief, LNPPS served as a scaffold to strengthen the association between PDCD5 and p53, which competitively impaired the formation of the MDM2/p53 complex, therefore inhibiting MDM2‐mediated p53 ubiquitination and degradation. Given that ubiquitination‐induced proteasomal degradation mostly occurs in the cytoplasm,^38^ we hypothesised that LNPPS mediated the translocation of PDCD5 and p53 into the nucleus so that the complex was sequestered from proteasomal degradation. As expected, our results demonstrated that LNPPS could induce the nuclear translocation of the PDCD5/p53 complex, thereby assisting them in escaping from cytoplasmic proteasomal degradation and ultimately stabilising their proteins. Of note, since subcellular localisation is governed by various factors, such as dynamic assembly of lncRNA–ribonucleoproteins, higher order nuclear organisation and RNA structural motifs,^39^ the underlying mechanisms of nuclear translocation of the LNPPS/PDCD5/p53 complex are worthy of investigations in the future.

m^6^A modification, emerging as a key post‐transcription regulator of gene expression patterns, participates in various eukaryotic cellular processes, including circadian rhythm, external stimulus response and tumourigenesis. As a dynamic and reversible modification, the correct deposition of m^6^A is mediated by a multi‐protein machinery consisting of ‘writers’, ‘erasers’ and ‘readers’. Several studies have recently demonstrated the increase or decrease of m^6^A modification in BC.^40^, ^41^, ^42^, ^43^ In our study, the m^6^A level of LNPPS in BC cells was lower than that of the human urothelial cell line. Moreover, the enriched m^6^A modification of LNPPS induced by METTL3 resulted in a prolonged half‐life of LNPPS. Consistent with this finding, overexpression of ALKBH5 decreased m^6^A modification on LNPPS and facilitated its decay, suggesting a clear role of m^6^A methylation in LNPPS suppression of BC. Considering the biological importance of m^6^A dependent on specific m^6^A readers, we checked out the potential LNPPS‐binding proteins and found hnRNPA2B1 among them. As expected, the m^6^A site 1 of LNPPS was identified as the binding target of hnRNPA2B1. A recent study has shown that hnRNPA2B1 recognises the m^6^A sites of ILF3 and maintains its mRNA transcript stability.^44^ Collectively, these findings suggest that hnRNPA2B1 binds the m^6^A‐bearing LNPPS and may serve as an intermediary for LNPPS stability mediated by METTL3 and ALKBH5.

As an emerging star in cancer therapy, more and more lncRNAs have shown the potential to improve therapeutic efficacy and development of combination therapy in various cancers. Most studies focus on targeted silencing of oncogenic lncRNAs to hinder cancer progression, such as using antisense oligonucleotides or small synthetic molecules/peptides that block the association with lncRNAs and their specifically functional binding partners.^12^ Our present study identified LNPPS as a novel tumour suppressor with the ability to inhibit BC growth in vivo. In the future, we will continue with the LNPPS‐based targeted BC therapy, targeting genomic reprogramming in BC cells by a genome‐editing approach to insert tumour suppressor lncRNAs.

## 5 CONCLUSIONS

Our study reveals that m^6^A‐regulated LNPPS functions as a scaffold for PDCD5 and p53, blocking PDCD5 ubiquitination and competitively inhibiting MDM2‐mediated p53 ubiquitination, which promotes PDCD5/p53‐related cell apoptosis. This work provides insights into the pathophysiology and treatment of BC from a lncRNA perspective.

## CONFLICT OF INTEREST

The authors declare they have no conflicts of interest.

## Supporting information

Supporting Information

Supporting Information

Supporting Information

Supporting Information

Supporting Information

Supporting Information

Supporting Information

Supporting Information

Supporting Information

Supporting Information

Supporting Information
