# Supplementary material for: Characterisation of a novel transcript LNPPS acting as tumour suppressor in bladder cancer via PDCD5‐mediated p53 degradation blockage
Source: Clin Transl Med. 2022 Dec 28;13(1):e1149. doi: 10.1002/ctm2.1149 (PMC9797767; doi:10.1002/ctm2.1149)
Supplement: Supplementary file 1 — Supporting Information [file CTM2-13-e1149-s001.docx]

**Characterization of a novel transcript LNPPS acting as tumor suppressor in bladder cancer via PDCD5-mediated p53 degradation blockage**

Juan Li^1,2#^, Yifan Wang^1#^, Xinya Zhang^1^, Xuemei Yang^1,2^, Qiuchen Qi^1,2^, Qi Mi^1^, Maoxiao Feng^1^, Yunshan Wang^1,2,3^, Chuanxin Wang^1,2,3*^, Peilong Li^1,2*^ and Lutao Du^1,2,3*^

**Supplementary Figures Legends**

**Figure S1. The identification of candidate differently expressed lncRNAs in BC.** (A-I) The relative expression levels of Top 10 candidate differently expressed lncRNAs, except for LNPPS, by RT-qPCR in 27 pairs BC tissues and adjacent non-tumor tissues. p values are determined by Wilcoxon matched-pairs signed rank test (A): ENST0000444286; (B): ENST0000610701; (C): ENST0000433108; (D): ENST0000624704; (E): ENST0000607600; (F): ENST0000625883; (G): ENST0000561241; (H): ENST0000576302; (I): ENST0000566974. (J) PCR amplification to verify the whole sequence of LNPPS using 5 pairs of gene-specific primers in 5637 cells. (K) The prediction of ORF and its BLAST results of these potential peptides by ORF Finder. (L) General information of LNPPS coding ability by Coding Potential Assessment Tool (CPAT, http://lilab.research.bcm.edu/). (M) The prediction of putative peptide encoded by LNPPS using Coding Potential Caculator2 (CPC2, http://cpc2.gao-lab.org/). Upper: Frequency sequences of putative peptide. Lower: Summary of LNPPS coding probability. (N) The relative expression levels of LNPPS in BC and non-BC cell lines by qPCR, which normalized by subtracting the reference GAPDH threshold cycle (CT) values. (O) The cellular fractionation PCR of LNPPS showing that LNPPS was mainly located in the nucleus both T24 and UM-UC-3 cells. (P) The cellular distribution of LNPPS in non-BC cell lines by RNA FISH assays. Blue: DAPI, Red: Cy3-labeled LNPPS, 18s and U6 probes. 18s: cytoplasmic control, U6: nucleus control. All scale bars: 20μm. Related to Figure 1.

**Figure S2. The role of LNPPS in BC cells migration, invasion and cell cycle arrest.** (A and B) The effect of LNPPS overexpression on the migration (A) and invasion (B) ability of 5637 and J82 cells by Transwell assays. Left: Representative images. Right: Quantization of the numbers of migrating or invading cells. Scale bars: 100μm. (C and D) The effect of LNPPS knockdown on the migration (C) and invasion (D) ability of 5637 and J82 cells by Transwell assays. Left: Representative images. Right: Quantization of the numbers of migrating or invading cells. Scale bars: 100μm. (E) The effect of LNPPS overexpression on the cell cycle distribution of 5637 and J82 cells by flow cytometry. Left: Representative images. Right: Quantization of the percentage of cycle distribution. (F) The expression of CyclinD1 and p21 after overexpressed LNPPS or empty vector in 5637 and J82 cells. n.s.: (no significant) > 0.05, *p< 0.05, **p<0.01 and ***p<0.001. Related to Figure 2.

**Figure S3. The discovery of candidate LNPPS-binding proteins and the role of LNPPS in the ubiquitination of PDCD5.** (A) Representative image of stain-free gels which pulled down by biotin-labeled sense and anti-sense LNPPS probes in J82 cells lysates. Red box showed the major differential bands. (B) RNA pull-down assays followed by Western blotting analysis indicates that EIF5A and RAB10 could not be precipitated by biotin-labeled sense LNPPS probes in 5637 and J82 cell lysates. (C) Co-IP assays of endogenous PDCD5 ubiquitination in 5637 cells expressing LNPPS or empty vector under treatment with MG132. (D) The role of PDCD5-K20 site in the LNPPS-regulated ubiquitination of endogenous PDCD5 in 5637 cells. (E) The levels of PDCD5 protein after expressed the full-length LNPPS or a series of truncated LNPPS plasmids in 5637 cells. (F) Co-IP assays showing the effect of the full-length or a series of truncated LNPPS on the ubiquitination of endogenous PDCD5 in 5637 cells. (G) RIP assays with anti-Flag and anti-IgG antibodies in 5637 cells after expressed Flag-PDCD5^WT^ or Flag-PDCD5^K20^ plasmids. Upper: Agarose gel electrophoresis of RIP-qPCR products. Lower: Relative enrichment of LNPPS in wild-type or mutant PDCD5^K20^ by qRT-PCR. (H) The effect of LNPPS on the ubiquitination levels of wild-type PDCD5 or mutant PDCD5^K20^ which mutated the 1-30 a.a. region of PDCD5 but preserved its K20 site. n.s.: (no significant) > 0.05, *p< 0.05, **p<0.01 and ***p<0.001. Related to Figure 4 and Figure 5.

**Figure S4. LNPPS enhances p53 signaling in 5637 and J82 cells.** (A) KEGG pathway enrichment of differently expressed genes in LNPPS stable overexpressed 5637 cells compared with control group. (B) The overlap of p53 potential target genes in LNPPS-overexpression cells based on Fischer’s list (Oncogene 2017, 36: 3943–3956). (C) The RNA levels of p53 several apoptosis-related targets after LNPPS overexpression in J82 cells. (D) The RNA levels of p53 apoptosis-related targets after LNPPS knockdown in 5637 and J82 cells. (E) Western blotting analysis showing the role of p53 in the LNPPS-mediated BC cells apoptosis. (F) The DNA-binding ability of p53 in 5637 and J82 cells as determined by the Dual-luciferase reporter assays. The data was normalized to Renilla luciferase. Wt-p53RE, mt-p53RE and non-p53RE referred to the wild-type p53-response elements, mutated wild-type p53-response elements and empty vector without wild-type and mutated p53-response elements, respectively. (G) ChIP-qPCR assays showing the transcriptional activity of different mutant states of p53 in cells. NCI-H1299 (null p53), RT4 (wild-type p53). BAX 3’UTR and PUMA 3’UTR were the negative control for the binding sites in the promoters of BAX and PUMA, respectively. (H) The effect of LNPPS on the transcriptional activity of p53 against BAX and PUMA in 5637 and J82 cells by ChIP-qPCR assays. n.s. (no significant) > 0.05, *p< 0.05, **p<0.01 and ***p<0.001. Related to Figure 6.

**Figure S5. LNPPS regulates p53 ubiquitination in a PDCD5-dependent manner.** (A and B) The RNA levels of p53 after LNPPS overexpression (A) and knockdown (B) in 5637 and J82 cells. (C) Representative images of IHC staining of p53 in paraffin-embedded xenograft tumors from LNPPS stably overexpressed mice models. Scale bars: 250μm and 50μm, respectively. (D) The correlation between the expression of LNPPS and p53 in BC tissues. The levels of LNPPS and p53 were examined in tumor tissues from 30 BC patients by RT-qPCR and western blotting analysis, respectively. (E) Upper: p53 protein levels in J82 cells after expressed LNPPS or empty vector and then treated with or without CHX (100μg/ml). Below: Quantization of p53 degradation rate. (F) Western blotting analysis showing the p53 protein levels in J82 cells transfected with sgRNAs specific for LNPPS or nc-sgRNA and then treated with MG132 (10μM) or DMSO for 6 hours. (G) The effect of LNPPS on endogenous p53 ubiquitination in J82 cells. (H) The effect of PDCD5 on the LNPPS-regulated endogenous p53 ubiquitination and MDM2-p53 interaction in J82 cells. (I) Co-IP assays showing the role of LNPPS in the PDCD5-p53 interaction in LNPPS-overexpressing 5637 cells. (J) The interaction between PDCD5 and p53 was attenuated in LNPPS knockdown cells. (K) IF assays showing the co-localization between p53 and PDCD5 in LNPPS-overexpressed 5637 and J82 cells compared with control group. Red: Alexa555-labeled PDCD5. Green: Alexa488-labbed p53. Blue: DAPI. Scale bars: 20µm. (L) The correlation between the expression of p53 and PDCD5 in BC tissues. The levels of p53 and PDCD5 were examined in tumor tissues from 30 BC patients by western blotting analysis. (M) RNA pull-down assays showing the binding of LNPPS and p53 when PDCD5 was silenced in BC cells. n.s. (no significant) > 0.05, *p<0.05, **p<0.01 and ***p<0.001. Related to Figure 7.

**Figure S6. LNPPS mediates the nuclear accumulation of PDCD5 and p53 in BC cells.** (A and C) IF assays showing the effect of LNPPS on the cellular distribution of PDCD5 (A) and p53 (C) in BC cells. Red: Cy3-labeled LNPPS. Green: Alexa488-labbed PDCD5 or p53. Blue: DAPI. Scale bars: 20µm. (B and D) Western blotting analysis showing the effect of LNPPS overexpression on the cellular distribution of PDCD5 (B) and p53 (D) in 5637 and J82 cells. *p<0.05 and **p<0.01. Related to Figure 7.

**Figure S7. LNPPS blocks the MDM2-mediated p53 ubiquitination and degradation.** (A) The protein levels of p53 and PDCD5 in J82 cells after expressed indicated sets of plasmids. (B) The role of LNPPS in the MDM2-mediated endogenous p53 ubiquitination and the MDM2-p53 interaction in J82 cells. (C and D) The effect of LNPPS on the interaction between p53 and MDM2 by Co-IP assays in 5637 cells (C) and J82 cells (D). Cell lysates were subjected to IP by anti-p53 antibody or anti-MDM2 antibody, respectively. (E and F) IP assays showing hardly interaction between PDCD5 and MDM2. HEK293T cells were co-transfected with Flag-PDCD5 and HA-MDM2 plasmids and then subjected to IP assays by anti-Flag and anti-IgG antibodies (E), or anti-HA and anti-IgG antibodies (F). n.s. (no significant) > 0.05, *p<0.05 and **p<0.01. Related to Figure 7.

**Figure S8. Low LNPPS expression is independent on METTL14- and FTO-mediated m^6^A modification.** (A) The expression of LNPPS under treatment with 5-aza-dc (5μM) or DMSO for 4 days in T24 and UM-UC-3 cells. (B and C) The expression of LNPPS after treated with SAHA (2μM), NaB (2μM), RFGP966 (1μM) or ACY-1215 (4μM) for 24 hours in T24 (B) and UM-UC-3 (C) cells, respectively. (D-G) The correlation between LNPPS and METTL3 (D), ALKBH5 (E), METTL14 (F) and FTO (G) in 30 BC tissues. (H and J) Western blotting analysis showing the overexpression efficiency after transfected with METL14 (H) or FTO (J) plasmids in 5637 and J82 cells, respectively. (I and K) The expression of LNPPS after transfected with METTL14 (I) or FTO (K) plasmids for 24 hours, respectively. n.s. (no significant) > 0.05, *p< 0.05 and **p< 0.01. Related to Figure 8.

**Figure S9**. **The role of LNPPS in RT4 cells that harbor wild-type p53.** (A) The efficiency of LNPPS overexpression and knockdown following transfection of RT4 cells with its overexpression plasmid or CRISPRi system. (B) The effect of LNPPS overexpression or knockdown on the cell apoptosis of RT4 cells using flow cytometry. (C) The expression levels of p53 in the LNPPS-overexpressing and LNPPS-knockdown RT4 cells. (D) Ubiquitinaiton IP assays showing the effect of LNPPS on endogenous wild-type p53 ubiquitination in RT4 cells. (E) The protein levels of p53 and its targets RT4 cells after co-transfection with LNPPS-overexpression plasmid and PDCD5-specific siRNAs. (F) The roles of PDCD5 in LNPPS-regulated endogenous wild-type p53 ubiquitination, as well as the interaction of PDCD5-p53 and MDM2-p53 in RT4 cells. n.s.: (no significant) > 0.05, *p<0.05, **p<0.01 and ***p<0.001.
