# Supplementary figures and images for: Characterisation of a novel transcript LNPPS acting as tumour suppressor in bladder cancer via PDCD5‐mediated p53 degradation blockage
Source: Clin Transl Med. 2022 Dec 28;13(1):e1149. doi: 10.1002/ctm2.1149 (PMC9797767; doi:10.1002/ctm2.1149)

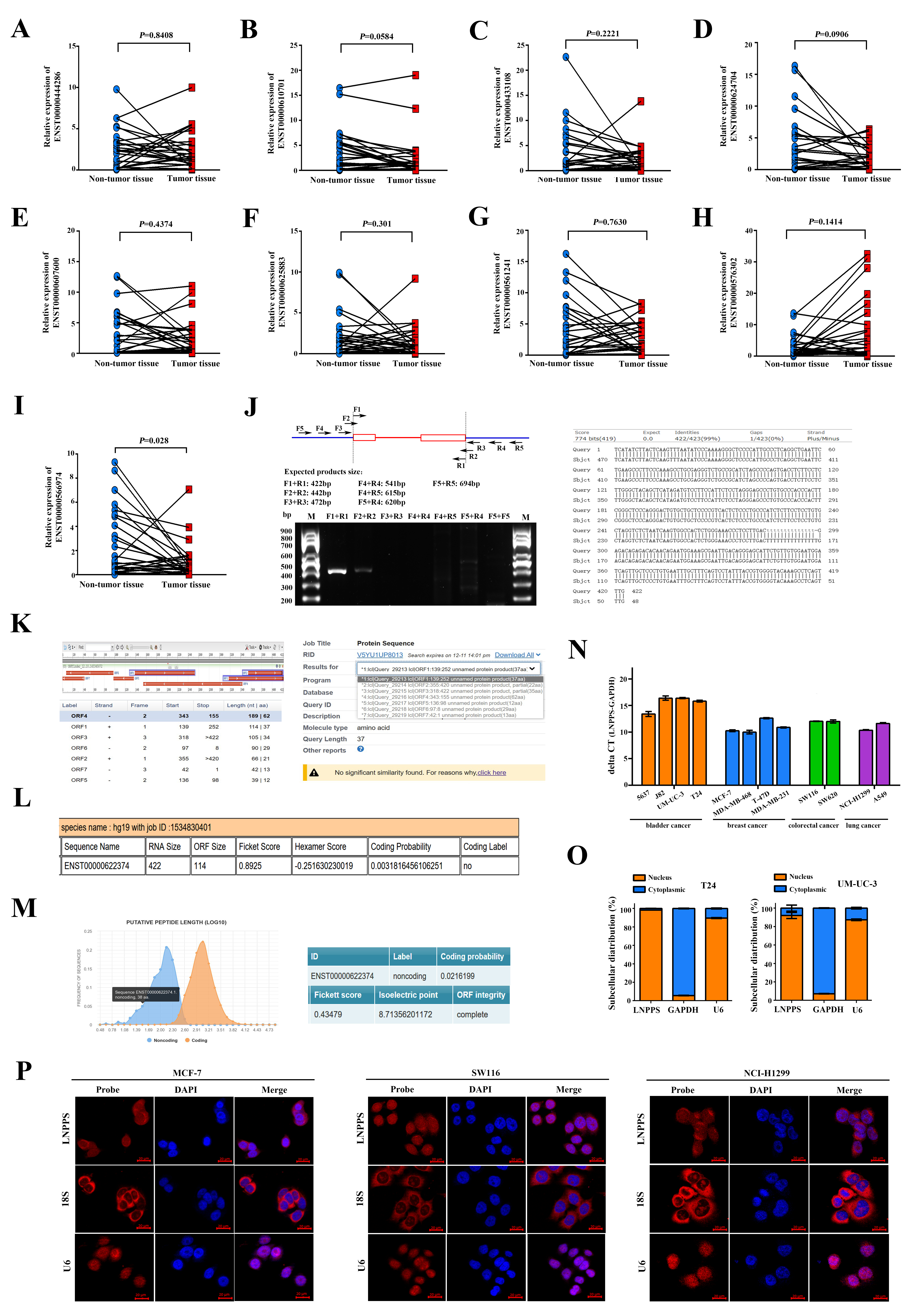

Supplement: Supplementary file 2 — Supporting Information [file CTM2-13-e1149-s004.tif]

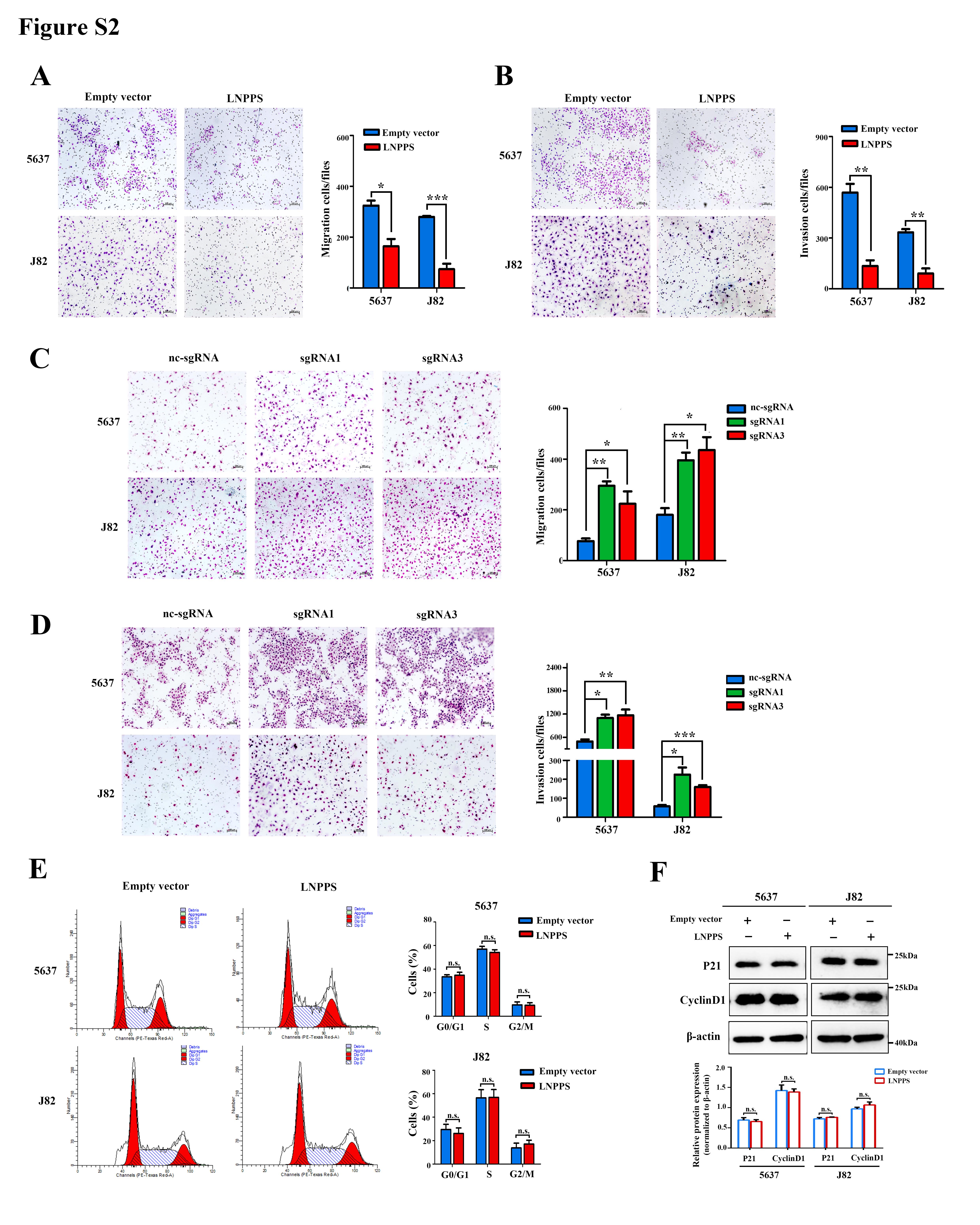

Supplement: Supplementary file 3 — Supporting Information [file CTM2-13-e1149-s006.tif]

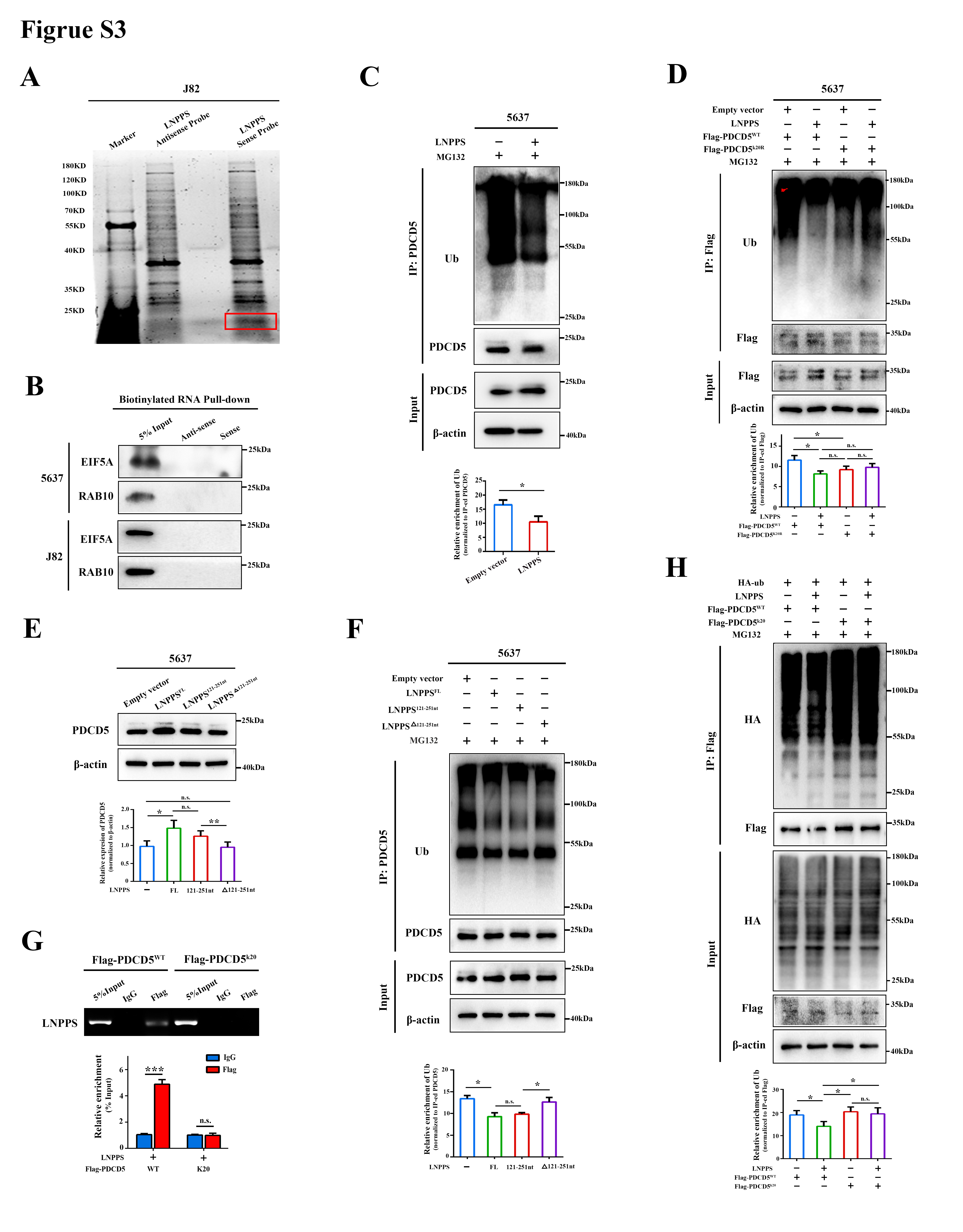

Supplement: Supplementary file 4 — Supporting Information [file CTM2-13-e1149-s010.tif]

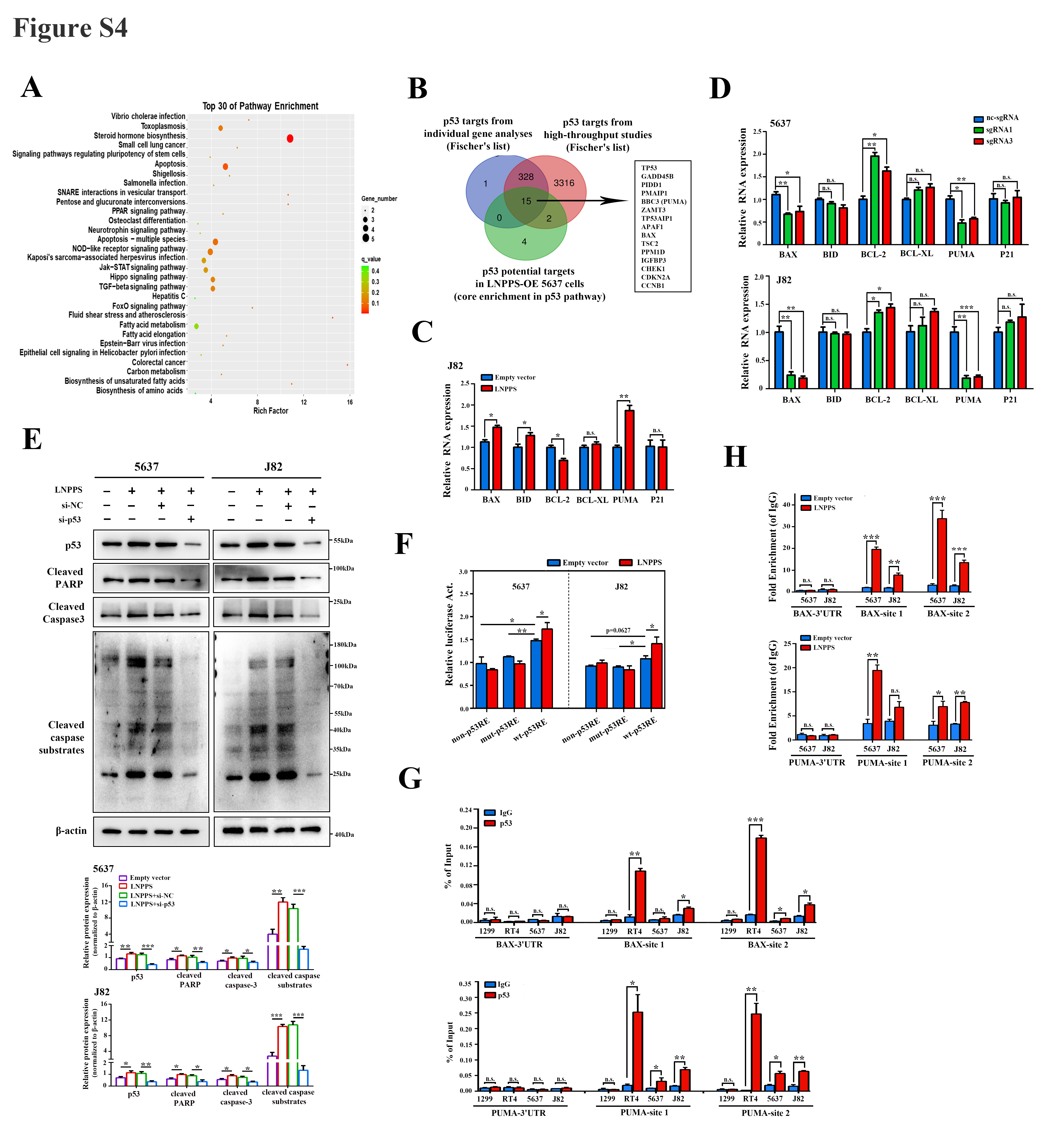

Supplement: Supplementary file 5 — Supporting Information [file CTM2-13-e1149-s011.tif]

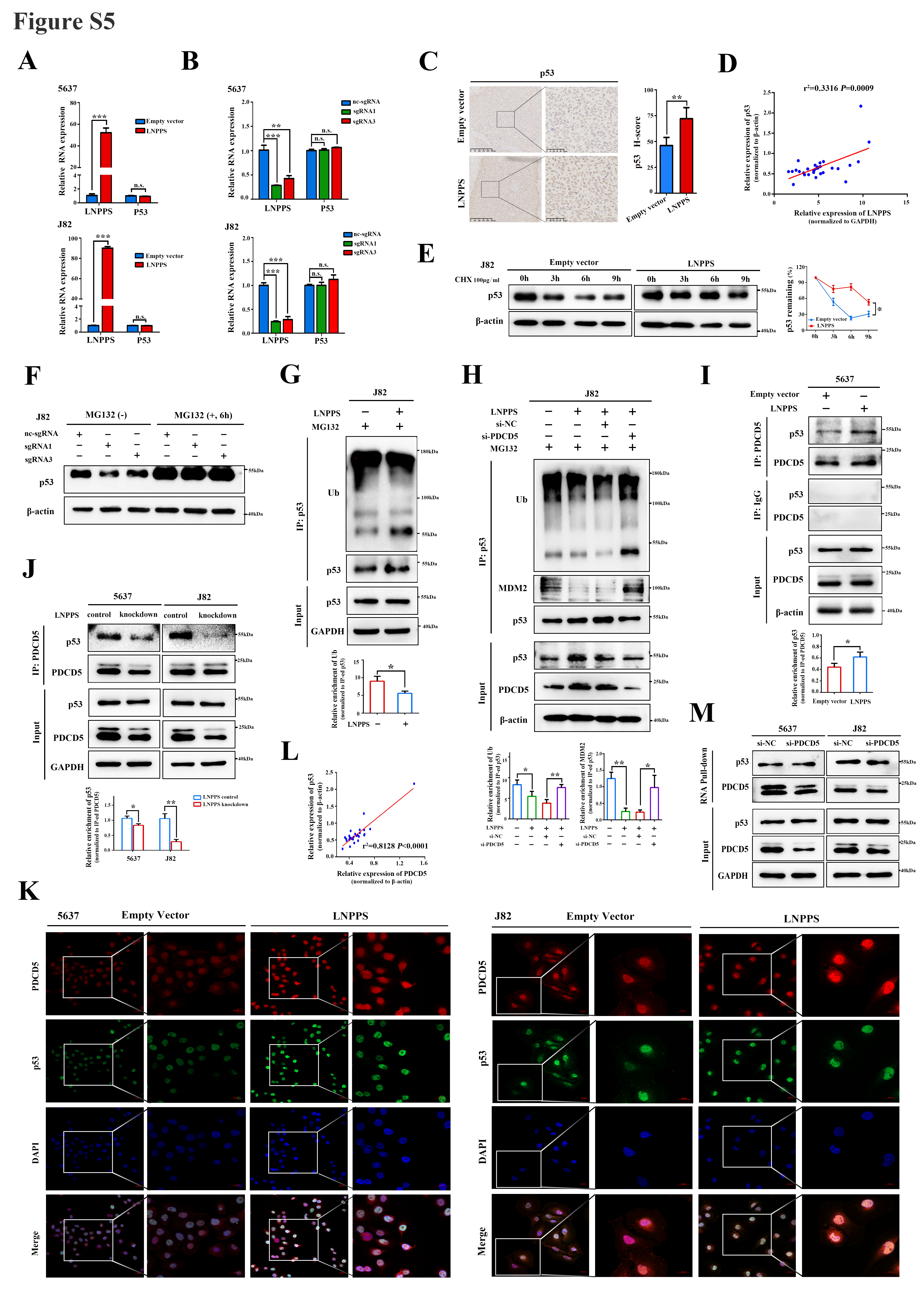

Supplement: Supplementary file 6 — Supporting Information [file CTM2-13-e1149-s005.tif]

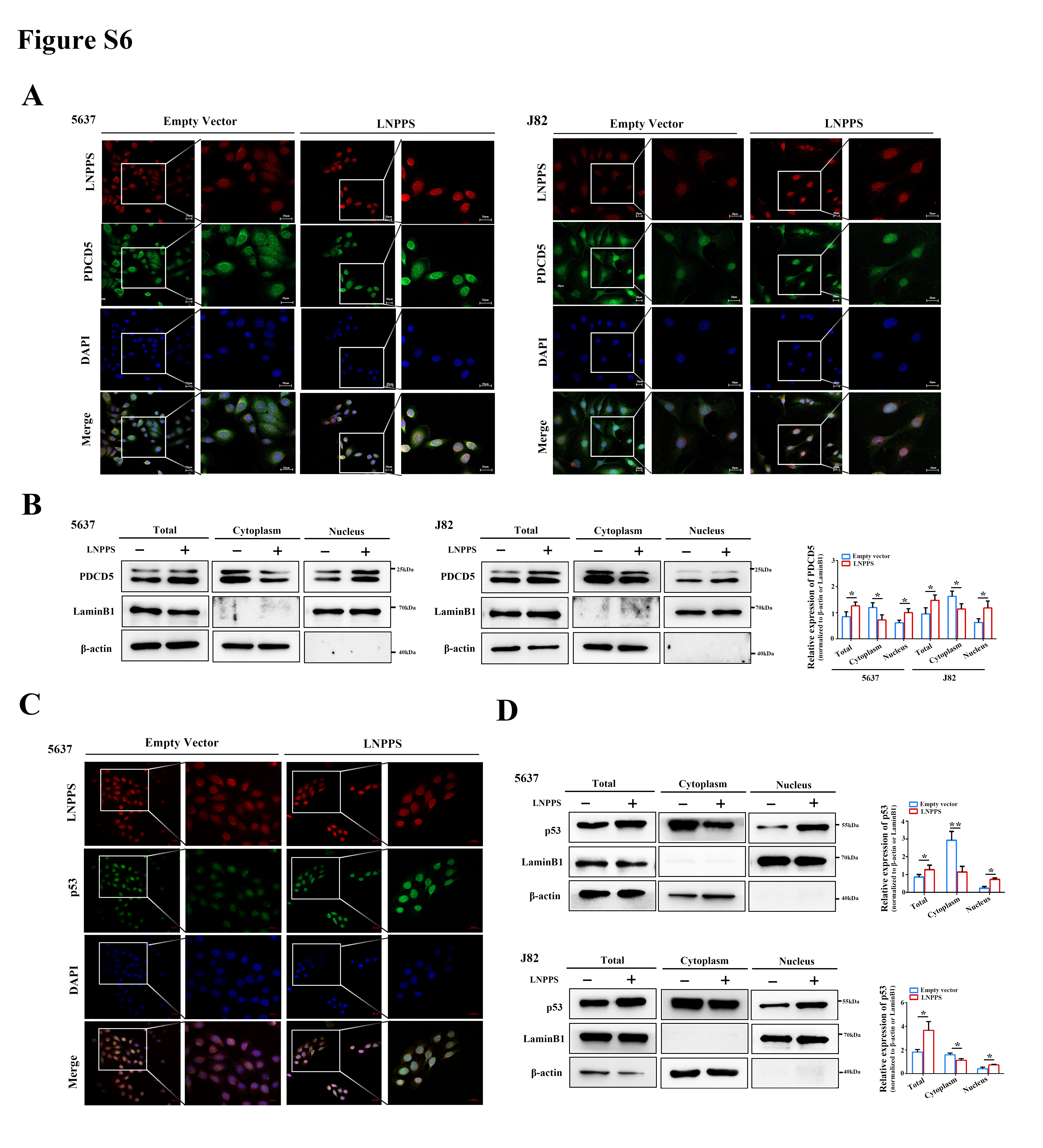

Supplement: Supplementary file 7 — Supporting Information [file CTM2-13-e1149-s007.tif]

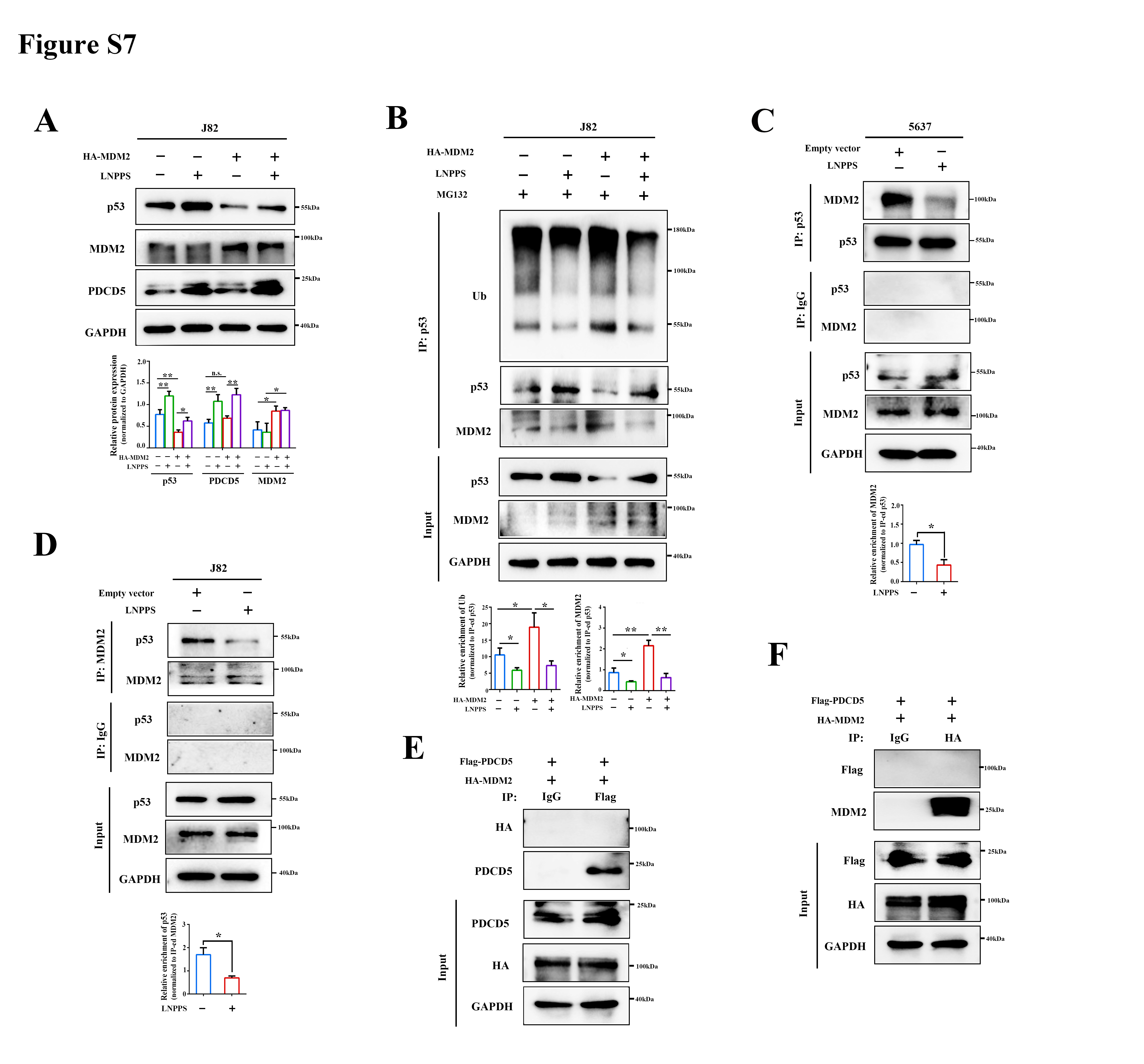

Supplement: Supplementary file 8 — Supporting Information [file CTM2-13-e1149-s002.tif]

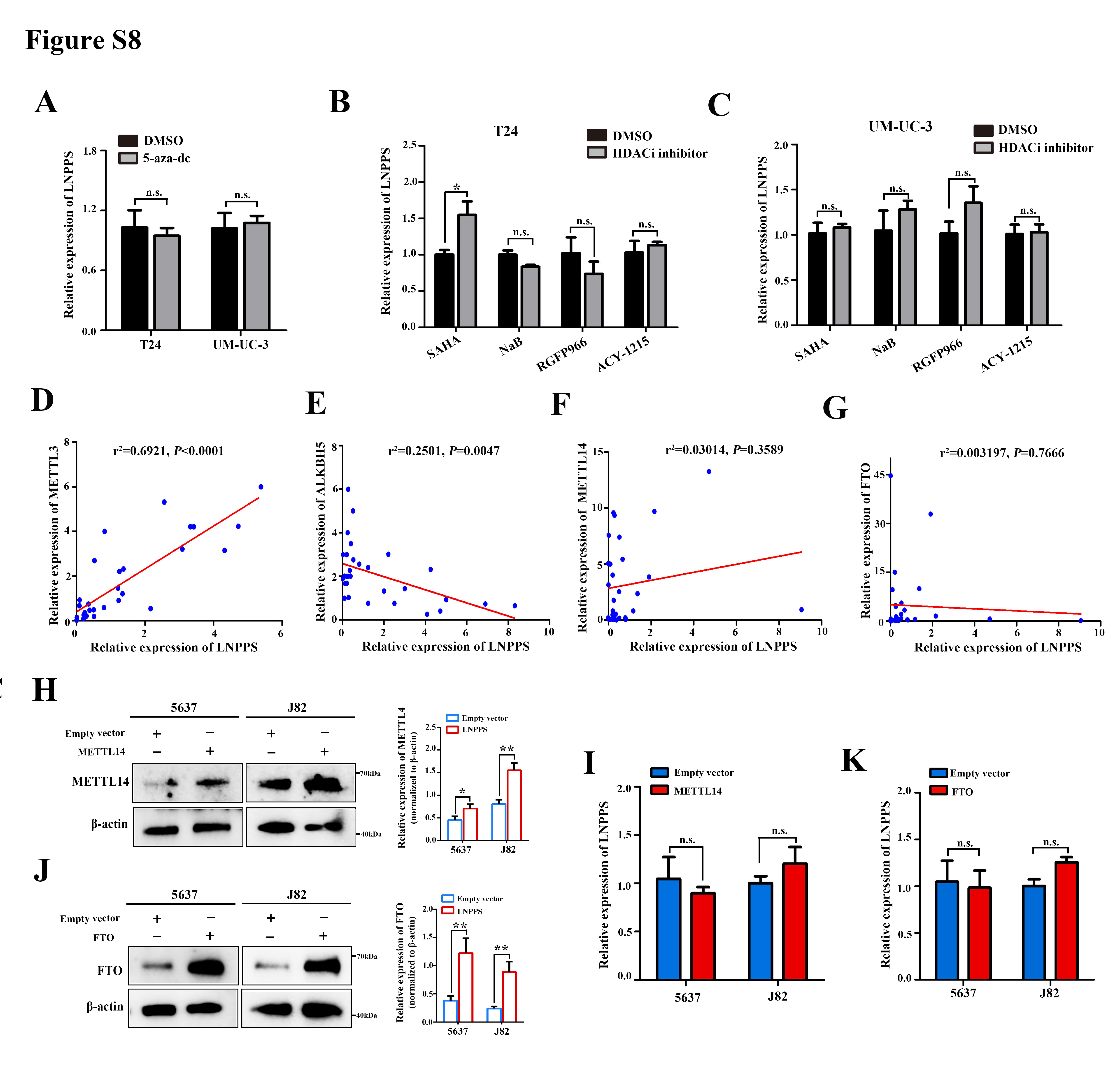

Supplement: Supplementary file 9 — Supporting Information [file CTM2-13-e1149-s003.tif]

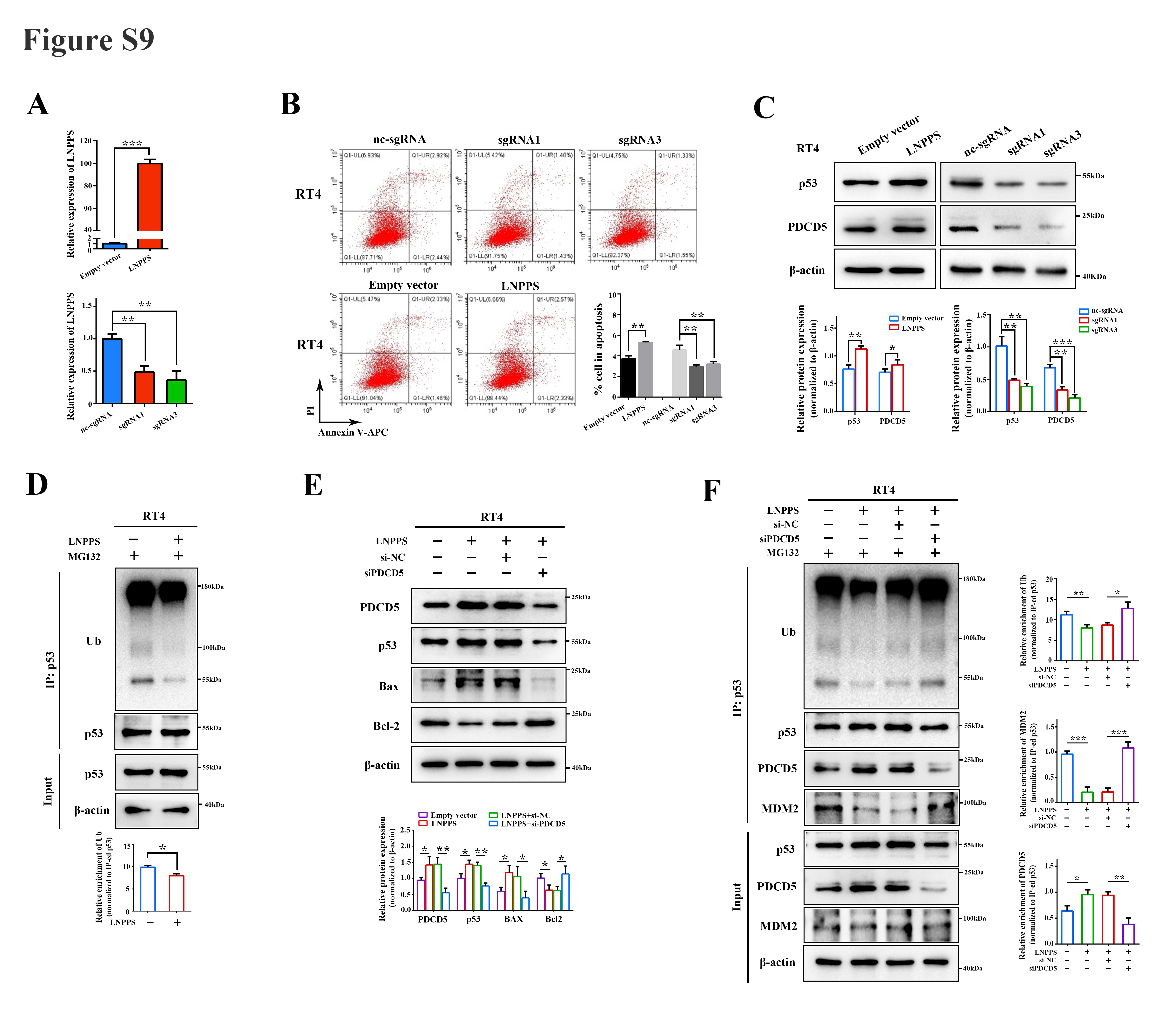

Supplement: Supplementary file 10 — Supporting Information [file CTM2-13-e1149-s009.tif]
