# Supplementary material for: Characterisation of a novel transcript LNPPS acting as tumour suppressor in bladder cancer via PDCD5‐mediated p53 degradation blockage
Source: Clin Transl Med. 2022 Dec 28;13(1):e1149. doi: 10.1002/ctm2.1149 (PMC9797767; doi:10.1002/ctm2.1149)
Supplement: Supplementary file 11 — Supporting Information [file CTM2-13-e1149-s008.docx]

**Characterization of a novel transcript LNPPS acting as tumor suppressor in bladder cancer via PDCD5-mediated p53 degradation blockage**

Juan Li^1,2#^, Yifan Wang^1#^, Xinya Zhang^1^, Xuemei Yang^1,2^, Qiuchen Qi^1,2^, Qi Mi^1^, Maoxiao Feng^1^, Yunshan Wang^1,2,3^, Chuanxin Wang^1,2,3*^, Peilong Li^1,2*^ and Lutao Du^1,2,3*^

**Supplementary Tables**

**Table S1.** The sequences of sgRNAs and siRNAs used in this study.

**Table S2.** The primers used in this study.

**Table S3.** The primary antibodies used in this study.

**Table S4.** Differently expressed lncRNAs in 3 pairs of BC and adjacent non-tumor tissues.

**Table S5.** Top 50 of candidate LNPPS-binding proteins identified by RNA pull-down assays.

**Table S6.** Differently expressed genes in LNPPS-overexpression 5637 cells compared with control group.

**Table S1. The sequences of sgRNAs and siRNAs used in the study**

| **ASOs/siRNAs** | **5’- Sequence -3’** |
| --- | --- |
| non-targeting control sgRNA | GTGTAGTTCGACCATTCGTG |
| LNPPS-sgRNA1 | CTAGGGGTTGTCTAAGGATA |
| LNPPS-sgRNA2 | GAATGCGCCAAGCATAAAGG |
| LNPPS-sgRNA3 | GGGGCATGGCATCAAAAGGT |
| LNPPS-sgRNA4 | CATGAATGATGCTGTTAGGC |
| si-PDCD5-1 | GGGCCAGGUUAAGUAACUUTT |
| si-PDCD5-2 | GCAAGAUAUGGACAACUAATT |
| si-p53-1 | GCACAGAGGAAGAGAAUCU |
| si-p53-2 | GAAAUUUGCGUGUGGAGUA |

**Table S2. The primers used in this study.**

| **Primers** | **Forward (5’-3’)** | **Reverse (5’-3’)** |
| --- | --- | --- |
| LNPPS | AATATCCCAAAAGGGCTCCCC | AAGAGGTCACTGGGGCTAGA |
| p53 | GCGCTTCGAGATGTTCCGAG | TATGGCGGGAGGTAGACTGAC |
| PDCD5 | GCCCAAGTTCTGGATCAGTCG | GTTGTCCATATCTTGCCATCTGT |
| METTL3 | TGATGCTGATCGACCCTGTC | CTTGGCGTGTGGTCTTTGC |
| METTL14 | GGCAGAAGTTACGGCGACAG | ATTTAACACGGCACCAATGCT |
| ALKBH5 | ATTAGATGCACCCCGGTTGG | TTGGGTTTCAGAGCAGGGTC |
| FTO | CTGCTCACTCCGGTATCTCG | GACCGTAAAGAGCCTGGTGT |
| LNPPS-m^6^A-site1 | CTGTGCCCACCCACTTCGG | GGAAGAGATGGGCAGGGAGAG |
| LNPPS-m^6^A-site2 | TGCTCCCCGTCACTCTCCC | AGTCAAGGAGGGTTTCCCAGAGT |
| P21 | TGTCTTGTACCCTTGTGCCT | TGGTAGAAATCTGTCATGCTGGTC |
| BAX | CTGACGGCAACTTCAACTGG | AGGAAGTCCAATGTCCAGCC |
| BID | GTCCTTGCTCCGTGATGTCTT | AGTCCATCCCATTTCTGGCT |
| Puma | CCCTGGAGGGTCCTGTACAAT | AATTGGGCTCCATCTCGGGG |
| BCL-2 | GGGTGAACTGGGGGAGGATT | ATCTCCCGGTTGACGCTCTC |
| BCL-XL | GGCAGCAGTAAAGCAAGCG | GCTCTGATATGCTGTCCCTGG |
| GAPDH | ACCCACTCCTCCACCTTTGAC | TGTTGCTGTAGCCAAATTCGTT |
| β-actin | AGAGCCTCGCCTTTGCCGAT | CCATCACGCCCTGGTGCCT |
| U6 | GCTTCGGCAGCACATATACTAAAAT | CGCTTCACGAATTTGCGTGTCAT |
| LNPPS-F1/R1 | TCATATCTTACTCAAGTTTAATATCCCAA | CAAACTGAGGCTTTGTACCCC |
| LNPPS-F2/R2 | TGCAAATATGCTCATATCTTACTCAAG | TGATAGCAGCAAACTGAGGCTT |
| LNPPS-F3/R3 | AATGATTTTTCAAGATGCAAATATGC | AAGGTGTCAAAGACATGATAGCAG |
| LNPPS-F4/R4 | TTTCTTCTGCCACTCCTCCTCTC | AAGCCTGGGCAATAAGTGAGAG |
| LNPPS-F5/R5 | ATTCCAGCCTAACAGCATCATTC | GTAACCATAAATACAGTAAAAATGAAACA |
| BAX-site 1 | AGGCTGAGACGGGGTTATCT | GCGCAGAAGGAATTAGCAAG |
| BAX-site 1 | GAAGGCTGAGACGGGGTTATC | TAGAAGTTTCGGGCAGGGTTT |
| BAX- 3’UTR | GCCTTGGACTGTGTTTTTCCTC | GTTTATTACCCCCTCAAGACCAC |
| PUMA-site 1 | GCGAGACTGTGGCCTTGTGT | CGTTCCAGGGTCCACAAAGT |
| PUMA-site 2 | ACTTTGTGGACCCTGGAACG | ACAGATCCACACCCCCAGC |
| PUMA-3’UTR | CGTGAAGAGCAAATGAGCCA | GCAGAGCACAGGATTCACAG |

**Table S3. The primary antibodies used in the study**

| **Antibodies** | **Source** | **Identifier** |
| --- | --- | --- |
| GAPDH | CST | 5174S |
| β-actin | CST | 8457 |
| LaminB1 | Proteintech | 12987-1-AP |
| cleaved caspase substrates | CST | 8698S |
| cleaved caspase-3 | CST | 9664S |
| cleaved PARP | CST | 5625S |
| PARP | Abcam | Ab32138 |
| PDCD5 | Abcam | ab83958 (low band is special for PDCD5) |
| p53 | Abcam | ab131442 |
| p53 | Santa | sc-126 |
| p53 | CST | 2527T |
| p53-ChIP Grade | Abcam | Ab1101 |
| MDM2 | Santa | sc-965 |
| p21 | Abcam | ab109520 |
| Cyclin D1 | CST | 2978S |
| BCL-2 | CST | 4223S |
| BAX | CST | 5023S |
| Ubiquitin | Santa | sc-8017 |
| Ubiquitin | Abcam | ab134953 |
| Flag-Tag | Sigma-Aldrich | F1804 |
| Flag-Tag | Abcam | ab205606 |
| HA-Tag | Abcam | ab49946 |
| HA-Tag | Abcam | ab9110 |
| METTL3 | CST | 96391 |
| METTL14 | CST | 51104 |
| ALKBH5 | Abcam | ab195377 |
| FTO | CST | 31687S |
| EIF5A | CST | 20765S |
| RAB10 | CST | 2187T |
| hnRNPA2B1 | Abcam | ab31645 |

**Table S4. Differently expressed lncRNAs in 3 pairs of BC and adjacent non-tumor tissues**

| **ID** | **log_2_\|fold change\|** | | **AveExpr** | **P.Value** | **adj.P.Val** |
| --- | --- | --- | --- | --- | --- |
| ENST00000561241 | -60.81 | 32.035 | | 5.82E-05 | 0.005124491 |
| ENST00000622374 | -20.01666667 | 10.43833333 | | 0.000860934 | 0.03178974 |
| ENST00000625883 | -30.52666667 | 18.96333333 | | 0.001083741 | 0.03178974 |
| ENST00000576302 | -29.48333333 | 22.67833333 | | 0.00357105 | 0.064964566 |
| ENST00000444286 | 55.35333333 | 28.58 | | 0.004473669 | 0.064964566 |
| ENST00000610701 | 23.99333333 | 27.14 | | 0.005503733 | 0.064964566 |
| ENST00000566974 | -164.6166667 | 83.62166667 | | 0.005879541 | 0.064964566 |
| ENST00000587434 | -173.69 | 101.3283333 | | 0.00590587 | 0.064964566 |
| ENST00000612252 | -16.88333333 | 12.29166667 | | 0.00874403 | 0.076283604 |
| ENST00000618132 | -573.7133333 | 653.4566667 | | 0.008801741 | 0.076283604 |
| ENST00000495094 | -31.45666667 | 18.67166667 | | 0.010090286 | 0.076283604 |
| ENST00000526310 | -26.14666667 | 18.62333333 | | 0.010811379 | 0.076283604 |
| ENST00000433108 | 34.81666667 | 17.795 | | 0.011269169 | 0.076283604 |
| ENST00000574246 | -11.40666667 | 10.44333333 | | 0.013579942 | 0.085359637 |
| ENST00000599781 | -8.393333333 | 10.04666667 | | 0.016412773 | 0.09628827 |
| ENST00000624704 | 11.48333333 | 13.28166667 | | 0.018325302 | 0.09929369 |
| ENST00000569274 | -12.65666667 | 12.61833333 | | 0.019181736 | 0.09929369 |
| ENST00000564363 | -49.55666667 | 25.49166667 | | 0.022188821 | 0.106614207 |
| ENST00000577048 | -161.6833333 | 81.915 | | 0.023018976 | 0.106614207 |
| ENST00000558905 | -179.3733333 | 91.73333333 | | 0.031446257 | 0.138363531 |
| ENST00000624094 | -105.6533333 | 57.18666667 | | 0.033406373 | 0.139988611 |
| ENST00000569467 | -20.54666667 | 15.01 | | 0.038262484 | 0.153049936 |
| ENST00000561409 | -9.45 | 11.23833333 | | 0.044932506 | 0.169297005 |
| ENST00000609955 | -10.9 | 12.77333333 | | 0.046302242 | 0.169297005 |
| ENST00000607600 | 24.59 | 17.54166667 | | 0.04809574 | 0.169297005 |

**Table S5. Top 50 of candidate LNPPS-binding proteins identified by RNA pull-down assay**

| **Accession** | **Description** | ΣCoverage | Σ#Proteins | Σ#Unique.Peptides | Σ# Peptides | Σ# PSMs |
| --- | --- | --- | --- | --- | --- | --- |
| **P63241** | **IF5A1** | **35.06** | **3** | **7** | **7** | **17** |
| Q5BLP8 | CD048 | 27.37 | 1 | 1 | 1 | 1 |
| Q5T1J5 | CHCH9 | 27.15 | 2 | 2 | 2 | 2 |
| **P61026** | **RAB10** | **26.5** | **19** | **2** | **4** | **8** |
| **O14737** | **PDCD5** | **26.4** | **1** | **3** | **3** | **6** |
| P56134 | ATPK | 25.53 | 1 | 2 | 2 | 4 |
| P62987 | RL40 | 25 | 3 | 1 | 4 | 13 |
| P84085 | ARF5 | 24.44 | 1 | 2 | 4 | 6 |
| P62306 | RUXF | 24.42 | 1 | 2 | 2 | 4 |
| P49458 | SRP09 | 23.26 | 1 | 2 | 2 | 2 |
| P14854 | CX6B | 20.93 | 1 | 1 | 1 | 1 |
| P07919 | QCR6 | 19.78 | 1 | 1 | 1 | 2 |
| Q02543 | RL18A | 18.18 | 1 | 3 | 3 | 6 |
| P63313 | TYB10 | 18.18 | 2 | 2 | 2 | 3 |
| P61960 | UFM1 | 17.65 | 1 | 1 | 1 | 1 |
| P08134 | RHOC | 17.62 | 1 | 1 | 3 | 6 |
| Q9Y547 | IFT25 | 17.36 | 1 | 1 | 1 | 1 |
| Q07812 | BAX | 16.67 | 1 | 2 | 2 | 2 |
| P62316 | SMD2 | 16.1 | 1 | 2 | 2 | 3 |
| P49773 | HINT1 | 15.87 | 1 | 1 | 1 | 1 |
| A8MWD9 | RUXG | 15.79 | 2 | 1 | 1 | 2 |
| P61088 | UBE2N | 15.79 | 2 | 2 | 2 | 3 |
| Q9NX14 | NDUBB | 15.03 | 1 | 1 | 1 | 1 |
| Q9Y5L4 | TIM13 | 14.74 | 1 | 1 | 1 | 2 |
| Q9P003 | CNIH4 | 14.39 | 1 | 1 | 1 | 1 |
| P37840 | SYUA | 14.29 | 1 | 1 | 1 | 1 |
| Q13185 | CBX3 | 13.66 | 1 | 2 | 2 | 2 |
| Q9BQ61 | CS043 | 13.64 | 1 | 1 | 1 | 1 |
| Q15370 | ELOB | 13.56 | 1 | 2 | 2 | 3 |
| O75396 | SC22B | 13.49 | 1 | 2 | 2 | 3 |
| P22914 | CRBS | 13.48 | 1 | 1 | 1 | 1 |
| Q8NBT2 | SPC24 | 13.2 | 1 | 1 | 1 | 1 |
| Q9NX55 | HYPK | 13.18 | 1 | 1 | 1 | 1 |
| O00233 | PSMD9 | 12.56 | 1 | 2 | 2 | 4 |
| Q13526 | PIN1 | 12.27 | 1 | 1 | 1 | 2 |
| P14406 | CX7A2 | 12.05 | 1 | 1 | 1 | 2 |
| O14684 | PTGES | 11.84 | 1 | 2 | 2 | 3 |
| Q7RTV0 | PHF5A | 11.82 | 1 | 1 | 1 | 1 |
| P20962 | PTMS | 11.76 | 1 | 1 | 1 | 1 |
| O95372 | LYPA2 | 11.26 | 1 | 2 | 2 | 4 |
| P61758 | PFD3 | 11.17 | 1 | 2 | 2 | 2 |
| P62314 | SMD1 | 10.92 | 1 | 1 | 1 | 1 |
| P51153 | RAB13 | 10.84 | 1 | 1 | 2 | 4 |
| P61923 | COPZ1 | 10.73 | 1 | 1 | 1 | 1 |
| O75964 | ATP5L | 10.68 | 1 | 1 | 1 | 1 |
| P00492 | HPRT | 10.55 | 1 | 2 | 2 | 4 |
| P09132 | SRP19 | 10.42 | 1 | 1 | 1 | 1 |
| P30046 | DOPD | 10.17 | 2 | 1 | 1 | 1 |
| P14927 | QCR7 | 9.91 | 1 | 1 | 1 | 1 |
| **Several proteins identified by RNA pull-down assay in sense-probe samples** | | | | | | |
| **Accession** | **Description** | **Σ**Coverage | **Σ**# Proteins | **Σ#** Unique.Peptides | **Σ#** Peptides | **Σ#** PSMs |
| P22626 | HNRNPA2B1 | 41.93 | 1 | 10 | 12 | 27 |
| O14737 | PDCD5 | 26.40 | 1 | 3 | 3 | 6 |
| P04637 | P53 | 8.14 | 1 | 3 | 3 | 4 |

**Table S6. Differently expressed genes in LNPPS-overexpression 5637 cells compared with control**

| **Gene_ID** | **Gene_Name** | **log_2_\|FC\|** | **P.Value** | **Gene_ID** | **Gene_Name** | **log2\|FC\|** | **P.Value** |
| --- | --- | --- | --- | --- | --- | --- | --- |
| ENSG00000275871 | AC131025.2 | 11.16 | 7.66E-82 | ENSG00000130311 | DDA1 | -0.83 | 6.57E-04 |
| ENSG00000125962 | ARMCX5 | -2.15 | 1.37E-24 | ENSG00000163635 | ATXN7 | 0.99 | 7.83E-04 |
| ENSG00000149257 | SERPINH1 | 1.05 | 3.85E-15 | ENSG00000267426 | AC087289.3 | -1.59 | 7.81E-04 |
| ENSG00000164080 | RAD54L2 | 1.13 | 1.07E-13 | ENSG00000139146 | SINHCAF | 0.87 | 7.94E-04 |
| ENSG00000282458 | WASH5P | -1.6 | 3.28E-11 | ENSG00000198218 | QRICH1 | 0.6 | 8.06E-04 |
| ENSG00000257184 | AC004080.3 | 2.59 | 3.67E-10 | ENSG00000158747 | NBL1 | 0.7 | 8.52E-04 |
| ENSG00000115738 | ID2 | -1.07 | 1.39E-09 | ENSG00000143882 | ATP6V1C2 | 0.77 | 1.07E-03 |
| ENSG00000183283 | DAZAP2 | 1.12 | 1.16E-08 | ENSG00000243646 | IL10RB | 0.78 | 1.04E-03 |
| ENSG00000116044 | NFE2L2 | 0.89 | 1.58E-07 | ENSG00000244687 | UBE2V1 | 0.79 | 1.06E-03 |
| ENSG00000179010 | MRFAP1 | -1.75 | 3.79E-07 | ENSG00000267532 | MIR497HG | -2.01 | 1.06E-03 |
| ENSG00000181029 | TRAPPC5 | -2.01 | 5.87E-07 | ENSG00000260108 | AC026464.2 | -3.95 | 1.14E-03 |
| ENSG00000171302 | CANT1 | -1.45 | 8.22E-07 | ENSG00000267368 | UPK3BL1 | -4.04 | 1.10E-03 |
| ENSG00000214736 | TOMM6 | -4.18 | 1.19E-06 | ENSG00000180089 | TMEM86B | 0.9 | 1.22E-03 |
| ENSG00000070087 | PFN2 | -0.97 | 3.21E-06 | ENSG00000272897 | AL109827.1 | 7.13 | 1.52E-03 |
| ENSG00000163159 | VPS72 | -0.77 | 3.17E-06 | ENSG00000169230 | PRELID1 | -0.67 | 1.97E-03 |
| ENSG00000213928 | IRF9 | 1.14 | 1.51E-05 | ENSG00000267436 | AC005786.3 | -6.89 | 2.00E-03 |
| ENSG00000184517 | ZFP1 | -0.86 | 2.42E-05 | ENSG00000166123 | GPT2 | -0.65 | 2.80E-03 |
| ENSG00000255730 | AC011462.1 | 7.04 | 4.51E-05 | ENSG00000197713 | RPE | -0.73 | 2.72E-03 |
| ENSG00000156875 | MFSD14A | 1.16 | 5.73E-05 | ENSG00000254692 | AL136295.1 | -6.1 | 2.78E-03 |
| ENSG00000153094 | BCL2L11 | -1.63 | 7.97E-05 | ENSG00000272578 | AP000347.1 | -0.82 | 2.82E-03 |
| ENSG00000149084 | HSD17B12 | -0.77 | 8.68E-05 | ENSG00000150991 | UBC | -1.25 | 2.94E-03 |
| ENSG00000138780 | GSTCD | 0.68 | 9.50E-05 | ENSG00000131467 | PSME3 | 0.94 | 3.22E-03 |
| ENSG00000256861 | AC048338.1 | 8.72 | 1.23E-04 | ENSG00000146112 | PPP1R18 | 0.63 | 3.17E-03 |
| ENSG00000109501 | WFS1 | 1.08 | 1.45E-04 | ENSG00000234545 | FAM133B | -0.77 | 3.16E-03 |
| ENSG00000100342 | APOL1 | 1.13 | 1.94E-04 | ENSG00000241978 | AKAP2 | -1.92 | 3.18E-03 |
| ENSG00000284292 | AC004922.1 | 1.63 | 2.20E-04 | ENSG00000260566 | AC127459.1 | -1.35 | 3.36E-03 |
| ENSG00000263244 | AC087190.3 | -4.23 | 2.32E-04 | ENSG00000272741 | AC069257.3 | -6.57 | 3.35E-03 |
| ENSG00000274049 | INO80B-WBP1 | -8.63 | 2.45E-04 | ENSG00000169255 | B3GALNT1 | 0.78 | 3.73E-03 |
| ENSG00000265735 | RN7SL5P | -2.16 | 2.57E-04 | ENSG00000126709 | IFI6 | 0.67 | 3.99E-03 |
| ENSG00000138760 | SCARB2 | -0.68 | 2.89E-04 | ENSG00000183354 | KIAA2026 | 0.71 | 4.13E-03 |
| ENSG00000197965 | MPZL1 | 1.14 | 3.05E-04 | ENSG00000260537 | AC012184.2 | 2.06 | 4.33E-03 |
| ENSG00000164284 | GRPEL2 | 1.27 | 3.31E-04 | ENSG00000080822 | CLDND1 | -0.86 | 4.57E-03 |
| ENSG00000136997 | MYC | 2.51 | 3.54E-04 | ENSG00000104899 | AMH | 0.7 | 4.62E-03 |
| ENSG00000053770 | AP5M1 | -0.69 | 3.71E-04 | ENSG00000106636 | YKT6 | -0.65 | 4.79E-03 |
| ENSG00000132603 | NIP7 | 0.88 | 3.72E-04 | ENSG00000124593 | AL365205.1 | 1.28 | 5.52E-03 |
| ENSG00000130479 | MAP1S | 1.07 | 4.33E-04 | ENSG00000143570 | SLC39A1 | 0.66 | 6.19E-03 |
| ENSG00000128050 | PAICS | 0.59 | 4.59E-04 | ENSG00000183479 | TREX2 | -0.82 | 6.27E-03 |
| ENSG00000143761 | ARF1 | 0.92 | 5.07E-04 | ENSG00000239969 | AC091390.4 | -1 | 6.31E-03 |
| ENSG00000171425 | ZNF581 | 0.7 | 5.12E-04 | ENSG00000240053 | LY6G5B | 0.74 | 6.50E-03 |
| ENSG00000205560 | CPT1B | 0.73 | 5.32E-04 | ENSG00000256966 | AL513165.2 | -1.12 | 6.59E-03 |
| ENSG00000178026 | LRRC75B | 0.76 | 6.18E-04 | ENSG00000196456 | ZNF775 | -0.91 | 6.96E-03 |
| ENSG00000151576 | QTRT2 | -0.83 | 6.40E-04 | ENSG00000175137 | SH3BP5L | -0.63 | 7.02E-03 |
| ENSG00000185414 | MRPL30 | -1.13 | 6.35E-04 | ENSG00000008086 | CDKL5 | -0.73 | 1.79E-02 |
| ENSG00000054793 | ATP9A | 0.62 | 7.62E-03 | ENSG00000225950 | NTF4 | 0.78 | 2.98E-02 |
| ENSG00000063978 | RNF4 | -1.07 | 9.71E-03 | ENSG00000226950 | DANCR | -0.69 | 4.58E-02 |
| ENSG00000073282 | TP63 | 0.62 | 4.01E-02 | ENSG00000228889 | UBAC2-AS1 | 0.64 | 3.30E-02 |
| ENSG00000077713 | SLC25A43 | -0.72 | 4.06E-02 | ENSG00000230638 | AL445933.1 | -0.73 | 2.93E-02 |
| ENSG00000089916 | GPATCH2L | 0.71 | 2.01E-02 | ENSG00000231439 | WASIR2 | -1.2 | 1.34E-02 |
| ENSG00000101745 | ANKRD12 | -0.68 | 2.84E-02 | ENSG00000232439 | RPL18AP7 | -1.23 | 2.01E-02 |
| ENSG00000101966 | XIAP | -0.86 | 1.24E-02 | ENSG00000234338 | AC073349.2 | -0.8 | 3.60E-02 |
| ENSG00000103502 | CDIPT | -0.61 | 8.83E-03 | ENSG00000234353 | AP000346.2 | 0.75 | 2.66E-02 |
| ENSG00000108219 | TSPAN14 | 0.81 | 2.56E-02 | ENSG00000235333 | PVRIG2P | -0.75 | 2.58E-02 |
| ENSG00000108861 | DUSP3 | -0.72 | 2.32E-02 | ENSG00000237436 | CAMTA1-DT | -2.04 | 1.21E-02 |
| ENSG00000112062 | MAPK14 | 0.73 | 3.82E-02 | ENSG00000237451 | CDK2AP2P2 | -1.04 | 4.73E-02 |
| ENSG00000119673 | ACOT2 | -0.62 | 2.21E-02 | ENSG00000240224 | UGT1A5 | 2.96 | 3.61E-02 |
| ENSG00000125449 | ARMC7 | -0.64 | 1.79E-02 | ENSG00000240963 | AL645465.1 | 2.35 | 2.80E-02 |
| ENSG00000131389 | SLC6A6 | 0.93 | 1.36E-02 | ENSG00000243225 | AC007686.1 | -1.23 | 3.38E-02 |
| ENSG00000132819 | RBM38 | -0.66 | 2.66E-02 | ENSG00000249007 | AL691482.3 | -1.5 | 1.46E-02 |
| ENSG00000135503 | ACVR1B | -0.68 | 8.36E-03 | ENSG00000249624 | AP000295.1 | -0.65 | 3.46E-02 |
| ENSG00000139112 | GABARAPL1 | -0.71 | 4.96E-02 | ENSG00000251349 | MSANTD3-TMEFF1 | -1.31 | 1.82E-02 |
| ENSG00000144580 | CNOT9 | 0.59 | 1.13E-02 | ENSG00000253816 | AC138866.1 | -0.93 | 2.48E-02 |
| ENSG00000149792 | MRPL49 | -0.89 | 4.88E-02 | ENSG00000256028 | AC026362.1 | 0.62 | 7.73E-03 |
| ENSG00000150779 | TIMM8B | -0.93 | 1.50E-02 | ENSG00000256514 | AP003419.1 | 1.13 | 3.81E-02 |
| ENSG00000151575 | TEX9 | -0.67 | 1.66E-02 | ENSG00000257122 | RRN3P3 | -0.66 | 1.60E-02 |
| ENSG00000155265 | GOLGA7B | -0.7 | 2.25E-02 | ENSG00000257950 | P2RX5-TAX1BP3 | 0.98 | 3.45E-02 |
| ENSG00000157224 | CLDN12 | 0.59 | 3.62E-02 | ENSG00000258232 | AC125611.3 | 1.61 | 1.32E-02 |
| ENSG00000160888 | IER2 | -1.04 | 4.82E-02 | ENSG00000259230 | LINC02323 | -0.77 | 3.83E-02 |
| ENSG00000161958 | FGF11 | 0.76 | 1.60E-02 | ENSG00000260238 | PMF1-BGLAP | 0.73 | 4.05E-02 |
| ENSG00000163472 | TMEM79 | -0.92 | 1.86E-02 | ENSG00000260272 | AC093525.2 | 0.83 | 2.13E-02 |
| ENSG00000168899 | VAMP5 | 0.77 | 1.48E-02 | ENSG00000260428 | SCX | 0.87 | 7.38E-03 |
| ENSG00000169499 | PLEKHA2 | -0.81 | 8.16E-03 | ENSG00000264772 | AC016876.2 | 1.14 | 1.02E-02 |
| ENSG00000170364 | SETMAR | 0.63 | 2.36E-02 | ENSG00000265315 | RN7SL199P | -1.57 | 2.52E-02 |
| ENSG00000170684 | ZNF296 | -0.59 | 2.46E-02 | ENSG00000265401 | AC093484.3 | 1.27 | 1.48E-02 |
| ENSG00000171161 | ZNF672 | -1.14 | 1.34E-02 | ENSG00000265800 | AC022211.3 | -0.9 | 3.26E-02 |
| ENSG00000173166 | RAPH1 | -0.78 | 3.30E-02 | ENSG00000267309 | AC092295.2 | -0.61 | 4.41E-02 |
| ENSG00000173875 | ZNF791 | 0.77 | 3.41E-02 | ENSG00000268533 | AC003002.2 | 1.31 | 2.69E-02 |
| ENSG00000175348 | TMEM9B | 0.59 | 2.12E-02 | ENSG00000270116 | AP001429.1 | -1.04 | 2.68E-02 |
| ENSG00000177576 | C18orf32 | -0.85 | 2.03E-02 | ENSG00000270419 | CAHM | -0.72 | 3.97E-02 |
| ENSG00000178295 | GEN1 | -0.77 | 1.95E-02 | ENSG00000271452 | AC005034.5 | -0.99 | 2.14E-02 |
| ENSG00000178761 | FAM219B | 0.65 | 2.64E-02 | ENSG00000271853 | AL162258.1 | -1.34 | 4.12E-02 |
| ENSG00000185480 | PARPBP | 0.59 | 1.19E-02 | ENSG00000271895 | AL109811.3 | 0.7 | 1.99E-02 |
| ENSG00000196436 | NPIPB15 | 0.69 | 2.24E-02 | ENSG00000272410 | AC022384.1 | -1.27 | 1.31E-02 |
| ENSG00000203362 | POLH-AS1 | -1.08 | 2.74E-02 | ENSG00000272910 | AC090425.2 | 1.17 | 4.56E-02 |
| ENSG00000205236 | AC105052.1 | -1.32 | 1.14E-02 | ENSG00000273184 | AC010655.4 | 0.82 | 1.69E-02 |
| ENSG00000206597 | SNORA57 | 1.4 | 3.13E-02 | ENSG00000273308 | AC024560.3 | -1.5 | 4.06E-02 |
| ENSG00000213082 | PPP1R14BP2 | -1.29 | 4.29E-02 | ENSG00000273723 | AL139089.1 | -0.84 | 3.70E-02 |
| ENSG00000225760 | LINC00431 | 0.97 | 2.67E-02 | ENSG00000274227 | AC073575.2 | 1.29 | 2.63E-02 |
| ENSG00000275202 | AL161421.1 | 0.71 | 3.82E-02 |  |  |  |  |
| ENSG00000275784 | AL034549.1 | -1.01 | 2.50E-02 |  |  |  |  |
| ENSG00000276168 | RN7SL1 | -1.51 | 1.74E-02 |  |  |  |  |
| ENSG00000277290 | AC136475.10 | -1.65 | 2.88E-02 |  |  |  |  |
| ENSG00000278000 | AC139100.2 | -1.16 | 1.20E-02 |  |  |  |  |
| ENSG00000278238 | AL359513.1 | 1.34 | 1.70E-02 |  |  |  |  |
| ENSG00000279809 | AC005538.2 | -1.06 | 1.82E-02 |  |  |  |  |
| ENSG00000283515 | AC020915.5 | -2.79 | 3.48E-02 |  |  |  |  |
| ENSG00000283761 | AC118553.2 | -1.25 | 1.46E-02 |  |  |  |  |
| ENSG00000284118 | MIR4707 | -3.35 | 8.29E-03 |  |  |  |  |
